# Collaborative metabolisms of urea and cyanate degradation in marine anammox bacterial culture

**DOI:** 10.1093/ismeco/ycad007

**Published:** 2024-01-10

**Authors:** Mamoru Oshiki, Emi Morimoto, Kanae Kobayashi, Hisashi Satoh, Satoshi Okabe

**Affiliations:** Division of Environmental Engineering, Faculty of Engineering, Hokkaido University, North 13, West 8, Kita-ku, Sapporo, Hokkaido 060-8628, Japan; Division of Environmental Engineering, Faculty of Engineering, Hokkaido University, North 13, West 8, Kita-ku, Sapporo, Hokkaido 060-8628, Japan; Division of Environmental Engineering, Faculty of Engineering, Hokkaido University, North 13, West 8, Kita-ku, Sapporo, Hokkaido 060-8628, Japan; Institute for Extra-Cutting-Edge Science and Technology Avant-Garde Research (X-star), Japan Agency for Marine-Earth Science and Technology (JAMSTEC), 2-15 Natsushima-cho, Yokosuka, Kanagawa 237-0061, Japan; Division of Environmental Engineering, Faculty of Engineering, Hokkaido University, North 13, West 8, Kita-ku, Sapporo, Hokkaido 060-8628, Japan; Division of Environmental Engineering, Faculty of Engineering, Hokkaido University, North 13, West 8, Kita-ku, Sapporo, Hokkaido 060-8628, Japan

**Keywords:** A marine anammox bacterial consortium, Scalindua sp, urea and cyanate degradation, collaborative metabolism

## Abstract

Anammox process greatly contributes to nitrogen loss occurring in oceanic oxygen minimum zones (OMZs), where the availability of NH_4_^+^ is scarce as compared with NO_2_^−^. Remineralization of organic nitrogen compounds including urea and cyanate (OCN^−^) into NH_4_^+^ has been believed as an NH_4_^+^ source of the anammox process in oxygen minimum zones. However, urea- or OCN^−^- dependent anammox has not been well examined due to the lack of marine anammox bacterial culture. In the present study, urea and OCN^−^ degradation in a marine anammox bacterial consortium were investigated based on ^15^N-tracer experiments and metagenomic analysis. Although a marine anammox bacterium, *Candidatus Scalindua* sp., itself was incapable of urea and OCN^−^ degradation, urea was anoxically decomposed to NH_4_^+^ by the coexisting ureolytic bacteria (*Rhizobiaceae*, *Nitrosomonadaceae*, and/or *Thalassopiraceae* bacteria), whereas OCN^−^ was abiotically degraded to NH_4_^+^. The produced NH_4_^+^ was subsequently utilized in the anammox process. The activity of the urea degradation increased under microaerobic condition (*ca.* 32–42 μM dissolved O_2_, DO), and the contribution of the anammox process to the total nitrogen loss also increased up to 33.3% at 32 μM DO. Urea-dependent anammox activities were further examined in a fluid thioglycolate media with a vertical gradient of O_2_ concentration, and the active collaborative metabolism of the urea degradation and anammox was detected at the lower oxycline (21 μM DO).

## Introduction

The global nitrogen cycle is a pivotal biogeochemical cycle on Earth, and massive nitrogen loss (N-loss) is occurring in the ocean, i.e. >240 Tg of nitrogen has been released in the form of N_2_ gas from the ocean into the atmosphere [1]. Oxygen minimum zones (OMZs) have been recognized as hotspots for oceanic N-loss, as 25%–35% of the N-loss occurs in the OMZs, even though the OMZs account for <1% of the ocean volume (when defined by dissolved O_2_, DO, ≤20 μM) [1-3]. The anaerobic ammonium oxidation (anammox) process, where NH_4_^+^ is oxidized to N_2_ gas using NO_2_^−^ as an electron acceptor [4], significantly contributes to oceanic N-loss occurring in the OMZs. For example, a previous survey of the Benguela and Eastern Tropical North Pacific OMZs showed that 30%–50% of N_2_ gas released from the OMZs has been produced through the anammox process [5-8]. Anammox bacteria are affiliated into a monophyletic group in the bacterial order *Brocadiales* of the phylum *Planctomycetota* [9-11], and the members affiliated into the genus *Candidatus Scalindua* are often regarded as marine anammox bacteria because most anammox bacterial 16S rRNA gene sequences retrieved from marine environments were affiliated into the *Scalindua* group [12, 13].

NH_4_^+^ availability becomes a rate-limiting factor of the anammox process occurring in oceanic OMZs [14-17] because NH_4_^+^ is often scarce in OMZ core water as compared with NO_2_^−^. For example, it is in the range of <1 μM in the Arabian Sea and a few μM in the Eastern Tropical South Pacific OMZ cores, respectively [18-21]. Remineralization of simple organic nitrogen compounds including coupled with microaerobic microbial respiration, denitrification, dissimilatory nitrite reduction to ammonium, and sulfate reduction can supply NH_4_^+^ for the anammox process [8, 15, 17]. Simple organic nitrogen compounds such as urea and cyanate (designated here as OCN^−^) are believed to be a source of NH_4_^+^ for the anammox process [8, 16, 21-24]. Indeed, the activities of anammox bacterial N_2_ gas production in oceanic OMZs were correlated with the availability of organic nitrogen compounds [16, 24, 25].

Urea is the primal product of the dissolved organic matter degradation and can be decomposed to CO_2_ and NH_4_^+^ enzymatically by urease (Ure), a Ni^2+^-containing metalloenzyme. Urea is excreted from zooplanktons and animals [23, 26], and available at a few 100 μM to submicromolar range in oxycline and OMZ core, respectively [21]. Other than urea, OCN^−^ is also available in oceanic OMZs (e.g. oxycline and OMZ cores of the Eastern Tropical South Pacific and Eastern Tropical North Pacific OMZs) [21, 23], whereas the concentration ranges of OCN^−^ were lower than those of urea (up to a only few 10 nM). OCN^−^ is produced from algal decomposition and from abiotic urea degradation [21, 27], and degraded to CO_2_ and NH_4_^+^ by cyanate hydratase (cyanase, Cyn) [28] and also by abiotic hydrolysis [29]. The occurrence of urea and/or OCN^−^-dependent anammox process in oceanic OMZs has been proposed in the early studies. The previous ^15^N-urea or -OCN^−^ incubation study using the marine water samples collected from the Eastern Tropical South Pacific OMZ revealed the production of the ^14-15^N_2_ gas derived from the anammox process [18]. However, the urea and ONC^−^ degradation by marine anammox bacteria have not been demonstrated so far, and it is yet to be examined whether *Scalindua* bacteria degraded urea and/or OCN^−^ or coexisting microorganisms were involved in the degradation. Furthermore, the gene sets involved in the urea or OCN^−^ degradation were not commonly found in the known *Scalindua* genomes, suggesting that the ability to degrade urea or OCN^−^ is not a common physiological trait of the *Scalindua* group. Therefore, cultivation-based analysis is definitely required to provide a direct evidence of urea and OCN^−^ degradation by *Scalindua* bacteria.

Therefore, the present study aimed to examine the degradation pathway of urea and OCN^−^ in a marine anammox bacterial consortium. Intriguingly, the marine annamox bacterium enriched from coastal sediment in Hiroshima Bay, *Candidatus Scalindua* sp. husus a7 (designated as *Scalindua* sp.), did not have both gene sets involved in the urea or OCN^−^ degradation, although their anammox activities were found when the *Scalindua* enrichment culture (>98% of total cells) was incubated with the addition of urea or OCN^–^ and NO_2_^−^. It is hypothesized that urea and OCN^–^ were degraded into NH_4_^+^ by coexisting bacteria and/or abiotically, and the produced NH_4_^+^ were sequentially utilized by the *Scalindua* sp. To test this hypothesis, the enrichment culture was anoxically incubated with the addition of urea or OCN^−^, and the activities of urea degradation, OCN^−^ degradation, and anammox process were carefully examined by ^15^N-tracing techniques. The incubation was repeated under different initial DO concentrations from *ca.* 5 to 42 μM to examine the influence of DO concentrations to the urea degradation. The urea degradation and anammox activities were further examined in a fluid thioglycolate media with a vertical gradient of O_2_ concentration (i.e. mimicking vertical zonation of O_2_ concentrations found in oxycline), and urea degradation and anammox bacterial growth were examined using the O_2_ and NH_4_^+^-selective microsensors and by determining *Scalindua* 16S rRNA gene copy number. Furthermore, ureolytic bacteria were enriched by subculturing with urea, and the enriched ureolytic bacteria were examined by (i) the amplicon sequencing of 16S rRNA gene, (ii) the amplicon sequencing of *ureC* encoding a catalytic subunit of Ure, and (iii) metagenomic analysis.

## Materials and Methods

### *Scalindua* biomass

Planktonic cells of *Scalindua* sp. obtained from a coastal sediment of the Hiroshima bay were enriched using a membrane bioreactor equipped with a hollow fiber membrane module (pore size 0.1 μm, polyethylene) as previously described [30]. The culture media fed into the membrane bioreactor (MBR) contained KH_2_PO_4_ (24.4 mg l^−1^), MgSO_4_ 7H_2_O (60 mg l^−1^), CaCl_2_ (51 mg l^−1^), yeast extract (Becton, Dickinson and Company, NJ) (1.0 mg l^−1^), an artificial sea salt SEALIFE (28 g l^−1^) (Marine Tech, Tokyo, Japan) [31], and 0.5 ml of trace element Solution I and II [32]. Equimolar amounts of NH_4_(SO_4_)_2_ and NaNO_2_ were supplemented into the media at 10 mM, and nitrogen loading rate of the MBR was set at 0.45 kg-N m^−3^ day^−1^. The MBR was operated at 25 °C in dark without pH control, but the pH was in the range of pH 7.6–8.0.

*Scalindua* sp. cells accounted for more than 90% of the total biomass in the MBR, which was further enriched by the buoyant density separation using Percoll solution (Cytiva) as previously described [33]. The Percoll-separated *Scalindua* biomass was washed with the culture media without NH_4_^+^ and NO_2_^−^ two times, and subjected to the following experiments. The abundance of *Scalindua* sp. cells in the Percoll-separated biomass increased to >98% of the total cells in the MBR, as determined by the fluorescence *in situ* hybridization analysis using the *Scalindua*-16S rRNA gene-specific Sca1129b oligonucleotide probe [31] (Supplementary Fig. S1).

### Activity tests

Standard anaerobic techniques were employed in an anaerobic chamber (Coy laboratories Products, Grass Lake, MI) where oxygen concentration was maintained at lower than 1 ppm. The culture media and stock solutions were prepared by purging N_2_ gas for >30 min, repeatedly vacuuming and purging He gas, and leaving in the anaerobic chamber for >1 week to remove trace amounts of oxygen dissolved in the media.

*Scalindua* biomass was resuspended in the above culture media without NH_4_^+^ and NO_2_^−^ at concentrations of 0.15–0.2 mg-protein ml^−1^, and 10–30 ml of the cell suspension was dispensed into 20–100 ml serum glass vials (Nichiden-Rika glass, Tokyo, Japan). The headspace was replaced with He gas (>99.99995%) after sealing with butyl rubber stoppers and aluminium caps. When the *Scalindua* biomass was incubated under microaerobic conditions, 0.5–20 ml of ambient air was injected into the headspace of the closed vials using a gas-tight syringe (GL Science) or disposable plastic syringe (Terumo). No air was injected for the anoxic incubations. To convert the volume of the injected air to the initial DO concentrations in the liquid media, the standard curve of the volume of the injected air (ml vial^−1^) versus the measured DO concentrations in the liquid media (μM) was prepared as previously describe by the authors [34]. Briefly, the vials without *Scalindua* biomass were incubated at 25 °C for 12 h after the air injection, and the DO concentrations in the liquid media were determined using an O_2_ microsensor (Unisense oxygen needle sensor OX-N 13621) (Unisense, Denmark). The initial DO concentrations were determined using the regression line of the prepared standard curve (Supplementary Fig. S2). The DO concentrations were not controlled during the microaerobic incubation, and more than half of the injected O_2_ remained in the head space at the end of the incubation.

The vials were incubated without the addition of substrate in the dark at 25 °C for 12 h, then the anoxic stock solution of the following substrate(s) was added at final concentration of 1–3 mM: Na^14^NO_2_^−^ (Fujifilm Wako), Na^15^NO_2_^−^ (^15^N content: >98 atom%) (Cambridge Isotope Laboratories, Andover, MA), ^14^N-urea (Fujifilm Wako), ^15^N-urea (>98 atom%) (Shoko Science, Kanagawa, Japan), Na^14^NCO (Fujifilm Wako). Penicillin G (final concentration; 500 mg/l) (Sigma Aldrich), or ATU (50 mg/l) (Fujifilm Wako) was supplemented to inhibit the activities of coexisting bacteria or aerobic ammonia oxidizers, respectively. The vials were incubated in dark at 25 °C, and liquid samples were collected using a 1-ml plastic disposable syringe, immediately filtered using 0.2-μm cellulose acetate filter, and subjected to determination of urea, OCN^−^, NH_4_^+^, NO_2_^−^, and NO_3_^−^ concentrations. Gas samples were collected using a gas-tight glass syringe and immediately injected to a gas chromatograph.

N-loss (μmol-N vial^−1^) from the liquid phase of the vials during the incubation was calculated using Equation (1)


(1) \begin{align*} &\left({N}_{urea}+{N}_{ammonium}+{N}_{nitrite}+{N}_{nitrate}\right)\nonumber\\&\quad-\left({N}_{urea}^{\ast }+{N}_{ammonium}^{\ast }+{N}_{nitrite}^{\ast }+{N}_{nitrate}^{\ast}\right) \end{align*}

where *N*: initial amounts of each nitrogen compounds fed into vials (μmol-N vial^-1^), N*: the amounts of each nitrogen compounds at the end of the incubation (μmol-N vial^−1^). The contribution of the anammox process to the N-loss was calculated by using Equation (2), where *N*_14–15*N*2_* (μmol vial^−1^) indicated the amounts of ^14–15^N_2_ gas produced by the end of the incubation.


(2) \begin{equation*} {N}_{14-15N2}^{\ast}\div nitrogen\ loss\times 100 \end{equation*}

### Chemical analysis

Urea concentration was determined as NH_4_^+^ concentration after enzymatic hydrolysis of urea to NH_4_^+^. Ure (Ure from Jack Bean, Fujifilm Wako) was dissolved in 10 mM PO_4_ buffer (pH 7.0) at a final concentration of 50 mg/l, and 5 μl of the Ure solution was added to 200 μl of liquid samples. After the incubation at 37 °C for 1 h, NH_4_^+^ concentration was determined colorimetrically or fluorometrically as described below.

OCN^−^ concentration was determined colorimetrically as previously described by Guilloton *et al*. [35]. Briefly, 0.5 ml of liquid sample was mixed with 0.5 ml of 10 mM anthranilonitrile solution, and incubated at 40 °C for 20 min. One milliliter of 12N HCl was added into the reaction mixture, and the mixture was further incubated at 99 °C for 5 min. After cooling down to room temperature, the absorbance was measured at 310 nm. Since NO_2_^−^ in the liquid sample increased the absorbance at 310 nm, NO_2_^−^ concentration was determined prior to OCN^−^ determination, and the absorbance derived from the coexisting NO_2_^−^ was then subtracted to calculate the OCN^−^ concentration specifically.

NH_4_^+^ concentration was determined colorimetrically by the indophenol method [36] and by fluorometrically using the *o*-phthalaldehyde (OPA) method [37]. Colorimetric determination was performed as described elsewhere. Briefly, liquid samples were mixed with phenol and hypochlorous acid solutions, and absorbance was measured at 635 nm. As for fluorometric determination, liquid samples were mixed with 3.8 mM OPA, and fluorescence intensity was determined at 355 nm of excitation and 460 nm of emission.

NO_2_^−^ concentration was determined colorimetrically using the naphthylethylenediamine method [36]. Liquid samples were mixed with a naphthylethylenediamine-sulfanilamide solution, and absorbance was measured at 540 nm.

NO_3_^−^ concentration was determined using an ion chromatograph IC-2010 equipped with the TSKgel SuperIC-Anion HS column (Tosoh, Tokyo, Japan).

^15^N atom percent of NO_2_^−^ was determined by matrix-assisted laser desorption ionization-time-of-flight mass spectrometry as previously described by the authors [38]. Briefly, liquid samples were mixed with naphthylethylenediamine reagent to develop NO_2_^−^-complex azo dyes, loaded onto a MALDI sample plate (MTP 384 target plate, ground steel BC, Bruker Japan, Yokohama, Japan) with α-cyano-4-hydroxy-cinnamic acid matrix. The peak-area ratios of *m/z* of 371 and 372 were determined using a Bruker Ultraflex III (Bruker Japan), and the ^15^N atom% was calculated using a calibration curve prepared using standard ^15^N-labeled compounds.

Protein concentration was determined by the Lowry method using a DC protein assay kit (Bio-Rad, Hercules, CA). Bovine serum albumin (Fujifilm Wako) was used as a protein standard.

Concentrations of ^14–15^N_2_ and ^15–15^N_2_ were measured by gas chromatography mass spectrometry [39]. About 50 μl of the headspace gas were collected using a 100-μl gas-tight glass syringe, and immediately injected into a gas chromatograph GCMS-QP 2010 SE (Shimadzu, Kyoto, Japan) equipped with a fused silica capillary column (Agilent Technologies, Santa Clara, CA). Peaks at *m/z* = 29 and 30 corresponding to ^14–15^N_2_ and ^15–15^N_2_ were monitored, and the concentrations of the N_2_ gas were calculated using a standard curve prepared using the ^15–15^N_2_ gas (Cambridge Isotope Laboratories).

### Enrichment of aerobic ureolytic bacteria

Thirty milliliter of the Percoll-separated *Scalindua* biomass was dispensed into a 100-ml glass vials with a silicon sponge plug (Silicosen, Shin-Etsu Polymer). After the addition of 1 mM ^14^N-urea, the vials were incubated at 25 °C without shaking (i.e. standing culture). Urea degradation in the culture was routinely monitored by determining urea, NH_4_^+^, NO_2_^−^, and NO_3_^−^ concentrations. After 32 days of the aerobic incubation, stock solution of ^14^N-urea was supplemented as a spike. After the depletion of the added ^14^N-urea, 10% of the culture was transferred into the fresh media containing 1 mM ^14^N-urea, and the incubation was repeated. This subculturing was repeated eight times, and the enrichment culture after 104 days of incubation was subjected to activity test, amplicon sequencing, and metagenomic analyses.

### Incubation of the *Scalindua* biomass in a fluid media

A fluid media was prepared as the conventional fluid thioglycolate media. The ^15^N-urea, ^14^NO_2_ (each 3 mM), 0.5 g/l Na_2_S, 1 mg/l resazurin, and 0.15% (w/v) gellun gum were added into the inorganic media (pH 7.5), and autoclaved after 30 min of N_2_ purge. About 50 ml of the autoclaved media was dispensed into a 69-ml glass vials in a clean bench (i.e. aerobic condition), and capped with butyl rubber cap and aluminum caps. The vials were incubated for 12 h without shaking at 25 °C to make a vertical O_2_ gradient inside of the media. The Percoll-separated *Scalindua* biomass (0.5 ml of the culture corresponding to 4 mg-protein) was inoculated onto the surface of the media using a gas-tight syringe and incubated at 25 °C for 28 days. After the incubation, DO and NH_4_^+^ concentrations in a fluid media were determined using the needle O_2_ microsensor and a liquid ion exchange membrane (LIX) microsensor, respectively [40]. The vial was uncapped under ambient air, and lifted up using a lab jack to reach the tip of the microsensor fixed on a clamp arm (Supplementary Fig. S3). The O_2_ microsensor was calibrated as following the instruction manual provided by the manufacture, and the sensor signals were recorded using a UniAmp Multi Channel. Ammonium-selective LIX microsensor was fabricated and calibrated as previously described by de Beer *et al.* [41]. The steady-state concentration profiles of DO and NH_4_^+^ in a fluid media were measured as previously described [40].

After the microsensor measurements, core samples were collected by inserting a sterile 10 ml plastic disposable pipette (Φ7.5 mm) (Supplementary Fig. S4) vertically into the fluid media and dispensed into a sterile 1.5 ml plastic centrifuge tube, which was subjected to DNA extraction using Fast DNA SPIN kit for Soil (MP Biomedicals). Quantitative PCR assay for determining *Scalindua* 16S rRNA gene copy number was carried out by *Taq*Man-based real-time PCR assay as previously described [42]. Premix Ex *Taq* (Takara Bio) and ABI prism 7500 sequence detection systems were used for the assay, and the standard curve for quantification was prepared by the 10-fold dilutions of plasmid DNA containing the *Scalindua* 16S rRNA gene molecule ranging from 10^7^ to 10^3^ copies μl^−1^.

### Amplicon sequencing of prokaryotic 16S rRNA gene and *ureC*

Genomic DNA was extracted from the Percoll-separated *Scalindua* biomass and the aerobic enrichment culture using a Lysis Solution F (Nippon Gene) and a bead-beading instrument (Shake Master Neo, BMS), and purified using MPure Bacterial DNA extraction kit (MP Bio) according to the manufacturer’s protocols. DNA concentrations were determined using Synergy LX (Bio Tek) and QuantiFluor dsDNA System. PCR amplification of prokaryotic 16S rRNA gene and *ureC* was performed using the oligonucleotide primer 515F (5′-GTGCCAGCMGCCGCGGTAA-3′)—806R (5′- GGACTACHVGGGTWTCTAAT-3′) [43] and L2F_V1 (5′-CGGCAAGGCCGGCAACCC-3′)—733R (5′- GTBGHDCCCCARTCYTCRT-3′) (amplicon size; *ca*. 291 and 386 bp for the 16S rRNA gene and *ureC*, respectively) [44] containing Illumina tag sequences at the 5′ ends, respectively. The PCR mixture had a volume of 10 μl and contained 4–100 ng of DNA, oligonucleotide primers (0.5 μM each), dNTPs (200 μM), 1× Ex polymerase chain reaction (PCR) buffer, and Ex*Taq* HS (0.05 U μl^−1^) (Takara Bio, Shiga, Japan). Cycling conditions were: 30 cycles at 94 °C for 30 s, followed by 50 °C for 30 s, then 72 °C for 30 s; and finally, 72 °C for 5 min. PCR products were purified using AMPure XP (Beckman Coulter) and tagged with sample-unique index and Illumina adapter sequences at their 5′ end by second PCR. The PCR reaction mixture (10 μl) contained 1–10 ng of the purified PCR products, oligonucleotide primers (0.5 μM each), dNTPs (200 μM), 1× Ex PCR buffer, and Ex*Taq* HS (0.05 U μl^−1^). Cycling conditions were: 10 cycles at 94 °C for 30 s, followed by 60 °C for 30 s, then 72 °C for 30 s; and finally, 72 °C for 5 min. Tagged amplicons were pooled, and sequenced by Illumina MiSeq platform in 300 bp paired-end sequencing reaction with v3 reagent kit (Illumina) according to the manufacturer’s instruction.

Sequence reads with the used oligonucleotide primer sequences were extracted using the fastx_barcode_splitter tool of the FASTX-Toolkit (ver. 0.0.14) (http://hannonlab.cshl.edu/fastx_toolkit/) and imported into the Qiime 2 (ver. 2021.4). In the Qiime 2 program [45], the removal of low-quality and/or chimeric sequence reads, as well as the removal of regions corresponding to the oligonucleotide primer and 50 bp of 3′ end, and the clustering of sequence reads into OTUs were carried out using the DADA2 plugin [46]. Phylogeny of the 16S rRNA gene OTUs was examined by the blastn (ver. 2.9.0) program using the Greengene (ver. 13_8) database, whereas the fungene (accessed on February 2023) and *nr* database (February 2021) were used for *ureC* OTUs.

### Metagenomic analysis

A shotgun sequence library was prepared from the Percoll-separated *Scalindua* biomass and the aerobic enrichment culture using an MGIEasy FS DNA Library Prep Set, MGIEasy Circularization kit, and DNBSEQ-G400RS High-throughput sequencing set (MGI Tech Japan, Tokyo, Japan). The 200-bp paired-end sequencing was performed using a DNBSEQ-G400 sequencer. The paired-end sequence reads were trimmed using Trimmomatic 0.39 (SLIDINGWINDOW:6:30 MINLEN:100) [47]. Assembled contigs were obtained using the Megahit v1.2.9 (--k-min 27 --k-max 141 --k-step 12) [48], and the contigs of short length (< 2,000 bp) were removed before binning. The contigs were subjected to binning analysis using the MaxBin2 version 2.15 (-markerset 40) [49], and the relative abundance of obtained bins was calculated using the CoverM version 0.6.1 (https://github.com/wwood/CoverM#installation). Phylogenetic position of each bin is estimated using GTDBtk v1.3.0 (release95) [50]. Gene prediction and annotation were performed via the D-FAST pipeline [51], and the MetaGeneAnnotator and Glimmer version 2.10, tRNAScan-SE version 1.23, and blastn software applications were used for the prediction of gene-coding sequences (CDSs), tRNA, and rRNA, respectively. Completeness and contamination were checked using CheckM version 1.0.7 [52].

### Accession numbers

Metagenomic sequence data and the sequence reads of 16S rRNA gene and *ureC* amplicons are available in the DDBJ nucleotide sequence database under the accession number DRA016463, DRA016464, and DRA016465, respectively.

## Results

### Urea and organic nitrogen compounds such as urea and cyanate degradation under anoxic condition

**Figure 1:**
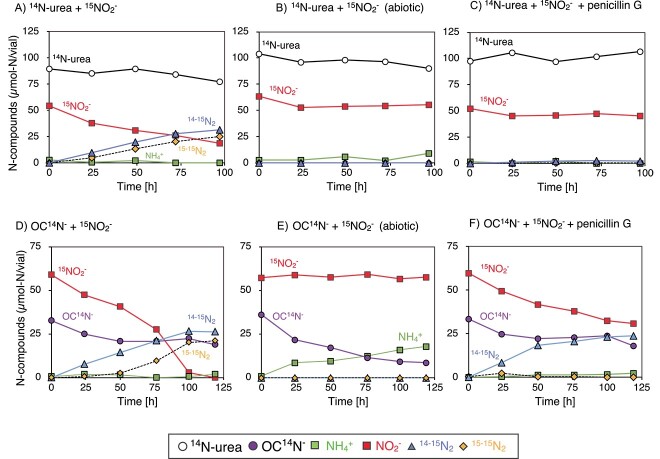
Anoxic urea and OCN^−^ degradation by the *Scalindua* biomass; 10 ml of the Percoll-separated *Scalindua* biomass was anoxically (<1 μM DO) incubated in 20-ml glass vials at 25 °C in dark with the addition of 3 mM ^14^N-urea and 3 mM ^15^NO_2_^−^ (A–C) or 3 mM OC^14^N^−^ and 3 mM ^15^NO_2_^−^ (D–F); the vials without *Scalindua* biomass were also prepared and incubated in parallel as abiotic controls (B and E); penicillin G was supplemented at a final concentration of 500 mg/l to inhibit the activities of coexisting bacteria (C and F); the incubations were performed in duplicate, and the plot symbols represented the mean values; the outcomes of the duplication incubations are shown in Supplementary Figs S5 and S6).

Highly enriched *Scalindua* biomass (>98% in total cells, Supplementary Fig. S1) was anoxically (<1 μM DO) incubated with the addition of (a) ^14^N-urea and ^15^NO_2_^−^ or (b) OC^14^N^−^ and ^15^NO_2_^−^ (each 3 mM). The consumption of ^15^NO_2_^−^ occurred concurrently with the production of ^14–15^N_2_ (Fig. 1A), which indicated that ^14^N-urea was degraded into ^14^NH_4_^+^, and the produced ^14^NH_4_^+^ and ^15^NO_2_^−^ were subsequently utilized for the anammox process. Similar behavior was found when OC^14^N^−^ was fed instead of ^14^N-urea (Fig. 1D). The rates of the ^14–15^N_2_ production in Fig. 1A and D were in the range of 0.1–0.2 μmol-N mg-protein^−1^ h^−1^, which was an order of magnitude lower than the maximum specific anammox activity for the *Scalindua* sp. [34]. Other than the ^14–15^N_2_ gas, ^15–15^N_2_ gas production also occurred during the incubation, which was likely produced via the denitrification of ^15^NO_2_^−^ (see below).

Occurrence of abiotic ^14^N-urea and OC^14^N^−^ degradations was examined by repeating the above anoxic incubation without the *Scalindua* biomass. The ^14^N-urea and ^15^NO_2_^−^ were not consumed, and no ^14–15^N_2_ and ^15–15^N_2_ gas was produced (Fig. 1B); therefore, the ^14^N-urea degradation was a biological process. On the other hand, the OC^14^N^−^ degradation occurred with NH_4_^+^ accumulation during the abiotic incubation with the rate of 3.41 μmol-N vial^−1^ day^−1^ (Fig. 1E). This abiotic OC^14^N^−^ degradation rate was comparable with the biotic OC^14^N^−^ degradation rate of the *Scalindua* biomass, i.e*.* 2.65 μmol-N vial^−1^ day^−1^ estimated from the ^14–15^N_2_ gas production rate in Fig. 1D. Therefore, abiotic degradation was responsible for the OCN^−^ degradation during the anoxic incubation.

The biological urea degradation by the *Scalindua* biomass was further investigated by adding the penicillin G which is an inhibitor of coexisting bacteria without inhibitory effects against anammox bacteria [53]. The addition of 500 μg/ml penicillin G resulted in no consumption of ^14^N-urea and ^15^NO_2_^−^ and no production of N_2_ gas (Fig. 1C), indicating that urea degradation was carried out by coexisting bacteria and not by *Scalindua* sp. When penicillin G was added with OC^14^N^−^ and ^15^NO_2_^−^, ^14–15^N_2_ gas but no ^15–15^N_2_ gas was produced (Fig. 1F). This result further supports that OC^14^N^−^ was degraded abiotically, and the produced ^14^NH_4_^+^ and ^15^NO_2_^−^ were converted into ^14–15^N_2_ gas by *Scalindua* sp., and the ^15–15^N_2_ gas (Fig. 1A and D) was produced via denitrification by coexisting bacteria. The absence of ^15–15^N_2_ gas production also indicates that *Scalindua* sp. did not dissimilatory reduce ^15^NO_2_^−^ to ^15^NH_4_^+^ [54, 55], and did not perform the anammox process using the formed ^15^NH_4_^+^.

### Urea degradation under microaerobic condition

**Figure 2:**
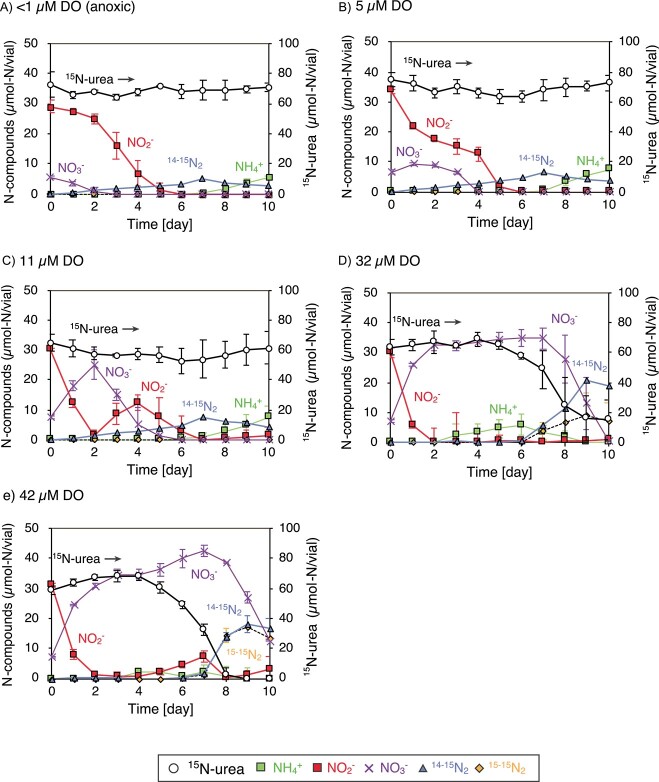
Urea degradation by the *Scalindua* biomass under anoxic and microaerobic conditions; 30 ml of the Percoll-separated *Scalindua* biomass was incubated in 100-ml glass vials at 25 °C in dark with the addition of 1 mM ^15^N-urea and 1 mM ^14^NO_2_^−^; (A) anoxic incubation without air injection; (B–E) ambient air was injected into the headspace of the vials to set the initial DO concentrations to be 5, 11, 32, and 42 μM; the symbols and error bars represent the mean value and the range of standard deviation derived from three biological replicates, respectively.

The *Scalindua* biomass was incubated under anoxic (<1 μM DO) and microaerobic conditions (*ca.* 5–42 μM DO) with the addition of ^15^N-urea and ^14^NO_2_^−^ to examine the influence of DO concentration on urea degradation. At <1, 5, and 11 μM DO, ^14–15^N_2_ gas was produced within 7 days at the rates of 0.57 ± 0.17, 0.86 ± 0.11, and 1.0 ± 0.23 μmol-N ^14–15^N_2_ vial^−1^, respectively (Fig 2A–C). The accumulation of NH_4_^+^ occurred after 6 days of the incubation without the production of ^14–15^N_2_ gas, which likely resulted from the depletion of NO_2_^−^ required for the anammox reaction. Both NO_2_^−^ and NO_3_^−^ (derived from the ground water used for the preparation of culture media) were consumed within 7 days of the incubation, whereas the ^14–15^N_2_ and ^15–15^N_2_ gas production were minor as compared with the NO_2_^−^ and NO_3_^−^ consumption. This difference indicated that the denitrification process producing ^14–14^N_2_ gas from NO_2_^−^ and/or NO_3_^−^ (NO_x_^−^) was responsible for the observed N-loss.

At 32 and 42 μM DO, urea degradation and ^14–15^N_2_ gas production greatly occurred after 6 days of incubation, with rates of 6.56 ± 0.26 and 8.11 ± 1.71 μmol-N ^14–15^N_2_ vial^−1^, respectively (calculated between 6 and 9 days and 7 and 9 days of the incubation) (Fig. 2D and E, respectively). These ^14–15^N_2_ gas productions occurred after the NH_4_^+^ or NO_2_^−^ accumulation period, indicating that NO_2_^−^ or NH_4_^+^ was the rate-limiting substrate at 32 and 42 μM DO, respectively. In addition to the ^14–15^N_2_ gas production, the production of ^15–15^N_2_ gas also occurred through both the anammox (^15^NH_4_^+^ and ^15^NO_2_^−^) and denitrification (^15^NO_x_^−^) process. The production of ^15^NO_2_^−^ from ^15^N-urea was examined by determining the ^15^N/^14^N atom ratio of the accumulated NO_2_^−^ at 42 μM DO. The ^15^N/^14^N atom ratio was >40 atom%, which was significantly higher than that of the NO_2_^−^ fed at the batch incubations (i.e. <5 atom%), indicating the production of ^15^NO_2_^−^.

The N-loss and the contribution of the anammox process increased when the initial O_2_ concentration was greater than 32 μM (Table 1). The highest N-loss and the greatest contribution of the anammox process were found at 32 μM O_2_, where 57.5 μmol-N/vial of N-loss and 33.3% of N-loss occurred via the anammox process. It is notable that the contribution was calculated from the amounts of ^14–15^N_2_ gas without ^15-15^N_2_ gas because the ^15–15^N_2_ gas could be produced through both the anammox and denitrification process as mentioned above. Thus, our calculation likely underestimated the contribution of the anammox process to the N-loss.

**Table 1:** N-loss and the contribution of anammox process under different initial DO concentrations.

DO (μM)	N-loss (μmol-N/vial)	Contribution of anammox
<1^a^	28.1 ± 7.3	9.8 ± 0.9%
5	31.0 ± 6.0	12.5 ± 3.8%
11	27.3 ± 4.5	16.1 ± 0.9%
32	57.5 ± 7.4	33.3 ± 3.9%
42	51.4 ± 4.4	32.0 ± 5.6%

About 30 ml of the *Scalindua* biomass was incubated in 100-ml glass vials under different initial DO concentrations with the addition of ^15^N-urea and ^14^NO_2_^−^ (each 1 mM). Behaviors of nitrogen compounds in liquid and gas phase are shown in Fig. 2. The values are the mean value and the range of standard deviation derived from three biological replicates.

a Anoxic incubation without air injection.

### Enrichment of aerobic ureolytic bacteria from the *Scalindua* biomass

**Figure 3:**
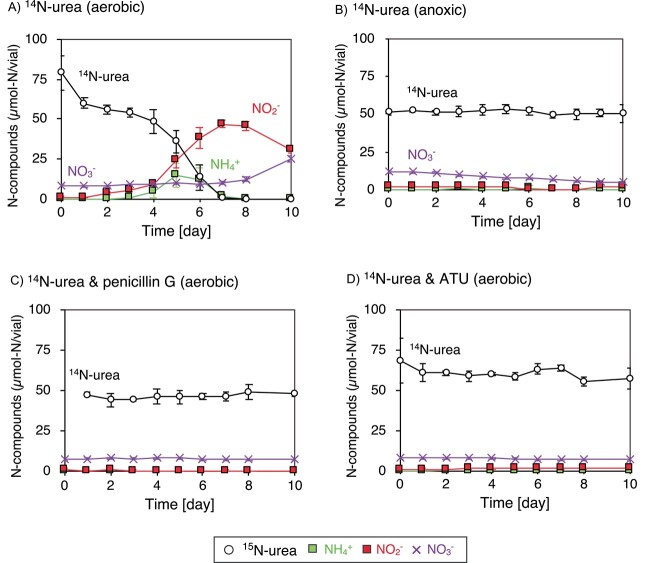
Urea degradation by aerobic enrichment culture; the Percoll-separated *Scalindua* biomass was subcultured eight times under aerobic condition with the addition of 1 mM urea as shown in Supplementary Fig. S5; 30 ml of the enrichment culture was dispensed into 100-ml glass vials with the addition of 1 mM ^14^N-urea and incubated under the following conditions: aerobic condition (A), anoxic condition (B), aerobic condition with penicillin G (C), and aerobic condition with ATU (D); the symbols and error bars represent the mean value and the range of standard deviation derived from three biological replicates, respectively.

Ureolytic bacteria were enriched by subculturing the *Scalindua* biomass with urea under aerobic condition. After 104 days of aerobic incubation with eight times subculturing, an enrichment culture showing high urea degradation activity was successfully obtained (Supplementary Fig. S7). Batch incubation of the enrichment culture was performed with the addition of urea under aerobic or anoxic conditions. The enrichment culture aerobically degraded the urea into NO_2_^−^ via NH_4_^+^, but the urea degradation did not occur under anoxic condition (Fig. 3A and B, respectively). The addition of 500 μg/ml penicillin G inhibited the ureolytic activities of the enrichment culture (Fig. 3C) as previously found in the *Scalindua* biomass (Fig. 1C). The accumulation of NO_2_^−^ during the above aerobic incubation (Fig. 3A) suggested that aerobic ammonia oxidizers capable of NH_3_ oxidation to NO_2_^−^ were present in the enrichment culture. Therefore, the above batch incubation was repeated by adding allylthiourea (ATU), an inhibitor of autotrophic aerobic ammonia oxidizers [56, 57]. ATU strongly inhibited the aerobic urea degradation, indicating aerobic ammonia oxidizers were responsible for the aerobic urea degradation (Fig. 3D).

### Urea degradation in oxycline and anoxic zone mimicking oxygen minimum zones

**Figure 4:**
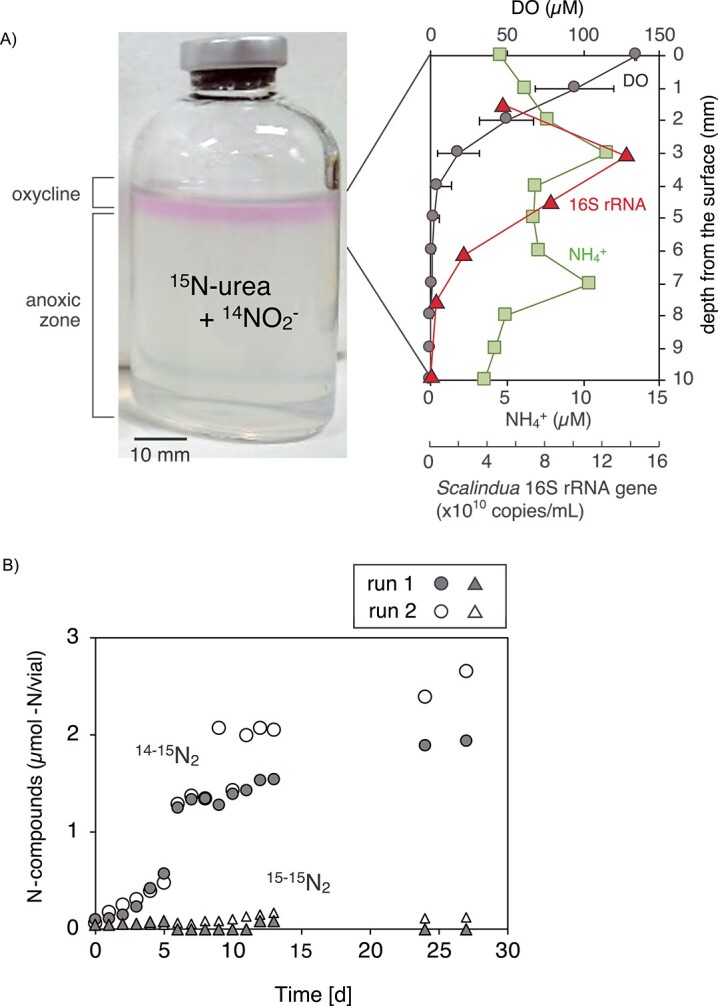
Urea degradation by the *Scalindua* biomass in a fluid thioglycolate media; (a) the fluid media containing 3 mM ^15^N-urea and 3 mM ^14^NO_2_^−^ was dispensed and solidified in a 69-ml closed glass vials with 19 ml of headspace air; vertical gradients of O_2_ concentrations were formed in the upper part of the fluid media by diffusion of air, which was visualized as a pink-colored band (the color of resazurin in the oxidized form) on the media; bar represents 10 mm of length; (B) vertical profiles of DO and NH_4_^+^ concentrations and abundance of the *Scalindua* 16S rRNA gene copy numbers after 28 days of incubation; 0 mm of depth corresponded to the surface of the fluid media; the symbols and error bars of the DO concentrations represent the mean value and the range of standard deviation derived from three biological replicates, respectively; as for NH_4_^+^ and *Scalindua* 16S rRNA gene copy numbers, the measurements were performed using one vial, and the error bar was not available; (c) production of ^14-15^N_2_ gas in the head space of the vials; the symbols and error bars of the DO concentrations represent the mean value and the range of standard deviation derived from three biological replicates, respectively.

Urea degradation of the *Scalindua* biomass was further examined using a fluid thioglycolate media with a vertical O_2_ gradient. The *Scalindua* biomass was incubated in a fluid media fed with ^15^N-urea and ^14^NO_2_^−^ (each 3 mM), and vertical profiles of DO and NH_4_^+^ concentrations were determined using the O_2_ and LIX-type NH_4_^+^ microsensors after 28 days of incubation, respectively. Oxycline was present to a depth of 5 mm, and the NH_4_^+^ concentration increased in the lower oxycline (Fig. 4A). The highest NH_4_^+^ concentration (11.5 μM) was found at a depth of 3 mm where DO concentration was 21 μM. The NH_4_^+^ concentration decreased in the bottom oxycline, and then the second NH_4_^+^ peak (10.4 μM) appeared at a depth of 7 mm. Anammox activities were examined by determining the production of the ^14–15^N_2_ gas in the headspace of the vials. The involvement of nitrification–denitrification process in the ^14–15^N_2_ gas production must be minor because the concentration range of the determined NH_4_^+^ was two orders of magnitude lower than the initial NO_2_^−^ concentration (i.e. <11.5 μM and 3 mM, respectively). A peak of the *Scalindua* 16S rRNA gene copy numbers was detected at 3 mm from the surface where the first NH_4_^+^ peak was detected, which indicated the occurrence of ^15^N-urea dependent anammox activities (Fig. 4B).

When the above incubation was repeated without the *Scalindua* biomass (i.e. abiotic incubation), no increase in NH_4_^+^ concentration was found in the fluid media (Supplementary Fig. S8A), indicating that urea degradation did not occur. Furthermore, there was no increase in the *Scalindua* 16S rRNA gene copy number, and no production of ^14–15^N_2_ and ^15–15^N_2_ gas was found when incubated with the addition of ^15^N-urea but not ^14^NO_2_^−^ (Supplementary Fig. S8B and C).

### Ureolytic bacteria in the *Scalindua* biomass

**Figure 5:**
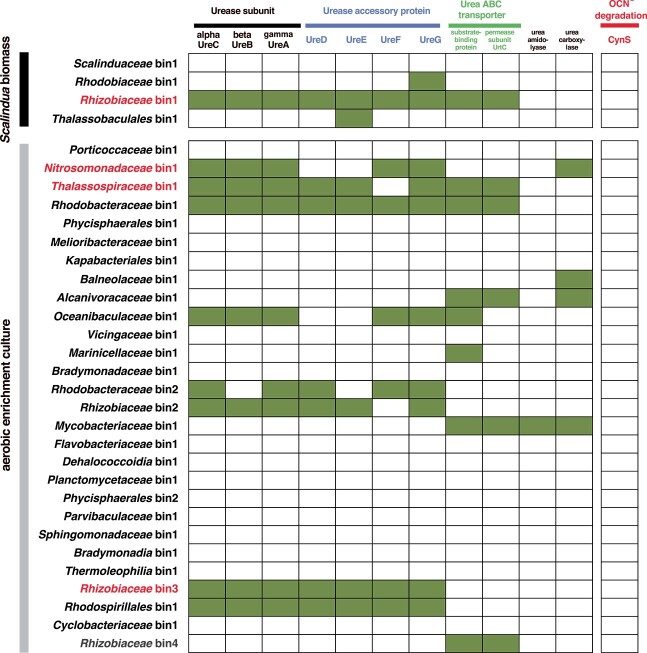
Presence/absence of the genes involved in biological ureolysis, urea transport, and OCN^–^ degradation on the metagenomic assembled bins obtained from the Percoll-separated *Scalindua* biomass and the aerobic enrichment culture; general features and phylogenies of the bins are available in Supplementary Table S1.

Amplicon sequencing analysis of prokaryotic 16S rRNA gene and *ureC* and also metagenomic analysis was carried out for the *Scalindua* biomass and the aerobic enrichment culture. In the *Scalindua* biomass, *Planctomycetota* (i.e. *Scalindua*) 16S rRNA gene was the most abundant (>97% of total reads), and the *Pseudomonadota*, *Cyanobacteriota*, *Chlorobiota*, *Bacteroidota*, and *Acidobacteriota* 16S rRNA genes were also detected (Supplementary Fig. S9). Four high-quality bacterial bins, *Scalinduaceae* bin1, *Rhodobiaceae* bin1, *Rhizobiaceae* bin1, and *Thalassobaculales* bin1 were obtained from the *Scalindua* biomass (Supplementary Table S1), and their metabolic potentials are summarized in Supplementary Fig. S10. As for the *Scalinduaceae* bin1, the gene sets involved in the anammox process and CO_2_ fixation via the Wood–Ljungdahl pathway were located in the bin (Supplementary Fig. S10). However, the gene sets required for urea degradation (i.e. *ureCBADEFG*, urea ABC transporter, urea amidolyase, and urea carboxylase) and also OCN^−^ degradation (*cynS* encoding Cyn) were absent in the *Scalinduaceae* bin1 (Fig. 5). This finding was consistent with the above outcomes of the ^15^N-tracer incubation (Fig. 1), i.e. coexisting bacteria other than *Scalindua* sp. and abiotic reactions were responsible for urea and OCN^−^ degradation, respectively. The gene sets involved in ureolysis were located in the *Rhizobiaceae* bin1 (*ureABCDEFG*, and urea ABC transporter), affiliated with the bacterial family *Rhizobiaceae*, but were not found in the *Rhodobiaceae* bin1 and *Thalassobaculales* bin1 (Fig. 5).

The ureolytic bacteria in the *Scalindua* biomass were further examined by the *ureC*-amplicon sequencing analysis. The *ureC* reads affiliated into the *Rhizobiaceae*, *Nitrosomonadaceae*, or *Thalassospiraceae* were found in the *Scalindua* biomass (Supplementary Fig. S11A). The *Rhizobiaceae ureC* reads were identical to the *ureC* sequence located in the above *Rhizobiaceae* bin1. The detection of the *Nitrosomonadaceae* and *Thalassospiraceae ureC* reads suggested that those bacteria other than *Rhizobiaceae* bacteria participated in the urea degradation of the *Scalindua* biomass. It should be noted, however, that the forward oligonucleotide primer used for the PCR amplification of *ureC* had some mismatched bases against the *ureC* sequence found on the obtained bins (Supplementary Fig. S11B); therefore, the abundance of the *ureC* reads shown in Supplementary Fig. S11A may not reflect the *in situ ureC* community structure due to PCR amplification bias.

In the aerobic enrichment culture, *Thalassospiraceae* (54.3% of total reads), *Nitrosomonadaceae* (12.9%), *Oceanospirillales*, and *Ignavibacteriales* 16S rRNA genes were abundant (Supplementary Fig. S9B), and 28 bacterial bins were obtained (Supplementary Table S1). Among the 28 bins, *Nitrosomonadaceae* bin1, *Thalassospiraceae* bin1, *Rhodobacteraceae* bin2, *Oceanibaculaceae* bin1, *Rhizobiaceae* bin2, *Rhizobiaceae* bin3, and *Rhodospirillales* bin1 had the *ureC* (Fig. 5). Among those bins, the *Nitrosomonadaceae* bin1, *Thalassospiraceae* bin1, and *Rhizobiaceae* bin3 *ureC* were identical with the *Nitrosomonadaceae*, *Thalassospiraceae*, and *Rhizobiaceae ureC* reads found in the above *ureC*-amplicon sequencing analysis (Supplementary Fig. S11A).

## Discussion

The present study is the first to describe the urea- and OCN^−^-dependent anammox activities in detail using a highly enriched marine anammox bacterial culture. Our ^15^N-tracer incubations have revealed that the collaborative metabolism of the urea degradation by coexisting bacteria and anammox bacteria occurred in the *Scalindua* biomass (Fig. 1A–C). The *Rhizobiaceae*, *Nitrosomonadaceae*, and *Thalassospiraceae* bacteria were the candidates of the ureolytic bacteria as examined by the *ureC* amplicon sequencing analysis (Supplementary Fig. S11A). Among those, *Nitrosomonadaceae* and *Thalassospiraceae* bacteria were successfully enriched in the aerobic enrichment culture, and the urea degradation of the enrichment culture only occurred under aerobic condition (Fig. 3). Therefore, the rest of the above candidates, *Rhizobiaceae* bacterium, was likely involved in the urea degradation of the *Scalindua* biomass under anoxic condition.

Ureolysis by the *Rhizobiaceae* bacteria has been demonstrated using some pure cultures (e.g. *Rhizobium galegae* and *Agrobacterium* strains) previously [58], while the *Rhizobiaceae* bin1 was affiliated into a phylogenetic clade without representative isolate. The closest relative of the *Rhizobiaceae* bin1 was the RICO01_sp004000235 bin, formerly obtained as dinoflagellates-associated bacterium, while the value of average nucleotide identity between the *Rhizobiaceae* bin1 was only 78.58, indicating that *Rhizobiaceae* bin1 represents a novel bacterial species; therefore, the metabolic capability of urea degradation by the *Rhizobiaceae* bin1 bacterium needs to be further investigated. As for the aerobic ureolytic bacteria, aerobic ureolysis was completely inhibited by adding ATU (Fig. 3). ATU is an inhibitor of copper-containing monooxygenases including ammonia monooxygenase and inhibits the growth of aerobic ammonia oxidizers including *Nitrosomonadaceae* [56]. *Nitrosomonadaceae* bacteria can degrade urea using Ure and oxidize the formed NH_4_^+^ to NO_2_^−^ sequentially [59, 60]; therefore, the aerobic ureolysis with NO_2_^−^ accumulation by the enrichment culture (Fig. 3) indicated that *Nitrosomonadaceae* but not *Thalassospiraceae* bacteria was responsible for the aerobic ureolysis. Notably, inhibition of urea degradation by aerobic ammonia oxidizers by ATU (Fig. 3D) has not been well characterized, and the questions have been raised as to whether ATU directly inhibited enzymatic activities of Ure, a Ni^2+^-containing metalloenzyme [61], or not.

In contrast to the biotic degradation of urea, OCN^–^ degradation occurred abiotically in the *Scalindua* biomass (Fig. 1). OCN^–^ can be degraded abiotically into NH_4_^+^ and CO_2_ via carbamic acid [29], and the abiotic degradation has been found in a sterile seawater [22]. Although the rate of abiotic OCN^−^ degradation (344 μmol-N l^−1^ day^−1^) (Fig. 1F) was 10^3^ order of magnitude higher than that previously determined using the seawater (0.463 μmol-N l^−1^ day^−1^ at 25 °C), it was reasonable by considering the initial OCN^−^ concentrations at the incubations (i.e. 3 mM and 10 μM, respectively) because the abiotic degradation rate was logarithmically dependent on the OCN^−^ concentration [29]. It should be noted that the OCN^−^ concentrations were found at the nanomolar range in the ocean [21], where the involvement of abiotic OCN^−^ degradation would be minor. Phylogenetically diverse bacteria including marine *Cyanobacteria* [22, 62] were capable of OCN^−^ degradation using Cyn, and they can contribute to OCN^−^ degradation and supply NH_4_^+^ for anammox process [18, 21].

Although *Scalindua* sp. itself was incapable of urea and OCN^−^ degradation, the genes encoding Ure (*ureC*) and/or Cyn (*cynS*) were located in some *Scalindua* bacterial genomes [23, 63-65] and those transcripts were found in the Eastern Tropical North Pacific and Eastern Tropical South Pacific OMZs [19], suggesting that some *Scalindua* bacteria are capable of direct degradation of urea and/or OCN^−^. Those metabolic capabilities would expand a habitat range of the *Scalindua* bacteria because the availability of NH_4_^+^ is typically scarce in OMZ core as compared with NO_2_^−^, and the remineralization of urea and OCN^−^ into NH_4_^+^ would be advantageous and increase their competitiveness. The *Scalindua* sp. examined in the present study has been enriched from a sediment of Hiroshima bay, where *in situ* concentrations of NH_4_^+^, NO_2_^−^, and NO_3_^−^ were determined to be 17.9, 2.9, and 6.4 μM, respectively [31, 66]. The NH_4_^+^ was not a limiting substrate of the anammox process in the sediment, and the metabolic capabilities of urea and OCN^−^ degradation were not beneficial as much as the *Scalindua* bacteria found in OMZ core; therefore, the *Scalindua* sp. genome did not have the gene sets involved in urea and OCN^−^ degradation. In the sense, the metabolic capability of urea and OCN^−^ degradation can define a niche of specific anammox bacteria. So far, nine anammox bacterial genera have been described in the order *Candidatus Brocadiales* (NCBI taxonomy database accessed on 13 November 2023), and different anammox species rarely coexisted in the culture [67, 68]. Several environmental factors including substrate concentrations and salinity (see the reviews, and the references in the review) [69, 70] have been identified as a driving force shaping the specific niche, whereas urea and OCN^−^ have not been recognized so far. It will be interesting to enrich anammox bacteria from environmental samples using urea or OCN^−^ instead of NH_4_^+^ as a substrate to examine whether urea or OCN^−^-degrading anammox bacteria can be enriched or not. Deeper understanding of urea and OCN^−^ metabolisms by anammox bacteria advances our knowledge of microbial ecology of anammox bacteria and their contribution to the global nitrogen cycle. In addition, the previous phylogenetic analysis of *Scalindua ureC* and *cynS* has suggested that those functional genes were acquired by lateral gene transfer [19, 23]. So far, genetic mechanisms of lateral gene transfer of anammox bacteria have not been well characterized, and mobile gene element (e.g. plasmid DNA) carrying Ure and Cyn has not been found from the anammox bacterial genomes. Lateral gene transfer of anammox bacteria is much of interest to further the understanding of the evolution and niche differentiation of anammox bacteria.

**Figure 6:**
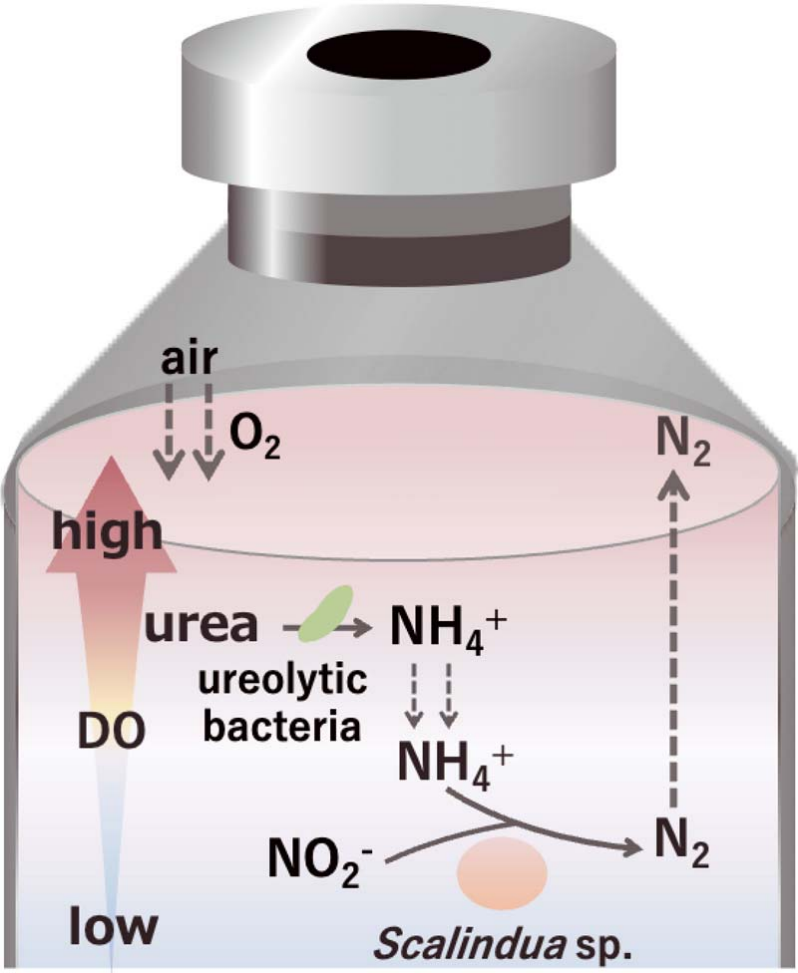
Substrate cross feeding from ureolytic bacteria to *Scalindua* sp. in fluid thioglycolate media; in the fluid thioglycolate media, a vertical gradient of DO concentration is available due to diffusion of O_2_ from atmosphere in the headspace of the vial; ureolytic bacteria degrade urea into NH_4_^+^, which is subsequently utilized by *Scalindua* sp. for the anammox process.

The incubation of the *Scalindua* biomass in the fluid thioglycolate media successfully produced a vertical O_2_ gradients in the media. The NH_4_^+^ production through the urea degradation and the growth of *Scalindua* sp. occurred concurrently in the lower oxycline (~21 μM DO) (Fig. 4), indicating a vertical partitioning of microbial activities with depth. Although anammox bacteria have been recognized as an obligatory anaerobic bacteria, the recent culture-based analyses have revised this understanding [71]. For instance, anammox bacteria and activities have been found from even microaerobic environments (<25 μM DO) [18, 72–74]. As for the *Scalindua* sp., this marine anammox bacterium has a genetic potential required for detoxification and tolerance for oxygen, and anammox activity has been found even under microaerobic condition [34]. The 50% inhibitory concentration and upper DO limits (*IC*_50_ and *DO_max_*, respectively) for the anammox activity of the *Scalindua* sp. have been determined to be 18.0 and 51.6 μM, respectively, and thus this anammox bacterium has been recognized as a microaerotolerant bacterium. In the lower oxycline, the *Scalindua* sp. depended on the *Rhizobiaceae*, *Nitrosomonadaceae*, and *Thalassospiraceae* bacteria for feeding NH_4_^+^ from urea degradation, and the possible collaborative metabolism occurred in the fluid media is shown in Fig. 6. In the oxycline, a variety of oxidation and reduction metabolisms can contribute to nitrogen conversion [15, 71]. When the *Scalindua* biomass was incubated at 5 and 11 μM DO, the reduction reactions (i.e. denitrification) were prominent, and the urea degradation into NH_4_^+^ was the rate-limiting step of the anammox activities (Fig 2B and C). In contrast, the oxidation reaction (NO_2_^−^ oxidation to NO_3_^−^) and urea degradation actively occurred under 32 and 42 μM DO, and the supply of NO_2_^−^ or NH_4_^+^ was the rate-limiting step of the anammox activities at 32 or 42 μM DO, respectively (Fig. 2D and E). Therefore, the fluctuation of DO concentration even in the narrow range significantly influenced on the metabolisms of nitrogen compounds.

In summary, a collaborative metabolism of the urea degradation by the *Rhizobiaceae*, *Nitrosomonadaceae*, and/or *Thalassospiraceae* bacteria and the anammox by the *Scalindua* sp. has been demonstrated under both the anoxic (<1 μM DO) and microaerobic (at 5–42 μM DO) conditions. In the case of OCN^−^, the abiotic degradation occurred under anoxic condition when OCN^−^ was fed at 3 mM. The presence of DO (32–42 μM) accelerated the occurrence of the urea-dependent anammox, and the oxic/anoxic interface became a hotspot of N-loss in the fluid media. Apart from the above collaborative metabolism, direct utilization of urea and OCN^−^ by some *Scalindua* bacteria is still required to be explored in other physiological studies using the *Scalindua* bacteria with *ureC* and/or *cynS*.

## Supplementary Material

SI_information_merged_ycad007

## Data Availability

Metagenomic sequence data and the sequence reads of 16S rRNA gene and *ureC* amplicons examined in the present study were available in the DDBJ nucleotide sequence database under the accession number DRA016463, DRA016464, and DRA016465, respectively.
