# Supplementary material for: Collaborative metabolisms of urea and cyanate degradation in marine anammox bacterial culture
Source: ISME Commun. 2024 Jan 10;4(1):ycad007. doi: 10.1093/ismeco/ycad007 (PMC10833080; doi:10.1093/ismeco/ycad007)
Supplement: SI_information_merged_ycad007 [file si_information_merged_ycad007.pdf]

Supplementary information

Collaborative metabolisms of urea and cyanate degradation in marine anammox bacterial culture

Mamoru Oshiki[1*], Emi Morimoto[1], Kanae Kobayashi[1, 2], Hisashi Satoh[1] & Satoshi Okabe[1*]

[1] Division of Environmental Engineering, Faculty of Engineering, Hokkaido University, North 13, West 8, Kita-ku, Sapporo, Hokkaido 060-8628, Japan

[2] Institute for Extra-cutting-edge Science and Technology Avant- garde Research (X-star), Japan Agency for Marine-Earth Science and Technology (JAMSTEC), 2-15 Natsushima-cho, Yokosuka city, Kanagawa, 237-0061, JAPAN

[*] These authors contributed equally: Mamoru Oshiki, Satoshi Okabe

Corresponding author:
Satoshi Okabe (Ph.D.)
E-mail; sokabe@eng.hokudai.ac.jp
Tel/Fax; +81-11-706-6266/7162

This manuscript contains 11 supplementary figure and 1 supplementary table. Those can be found in the online version of this article on the publisher's website.

Table S1 Summary of metagenomic bins obtained from the Percoll-separated *Scalindua* biomass (top) and the aerobic enrichment culture (bottom).

Bin	Relative abundance (%)	Size	Contigs	CDSs	Completeness	Contamination	Phylogeny
Scalinduaceae bin1	96.5%	4.3	516	3,330	95.8	8.3	p__*Planctomycetota*;c__*Brocadiae*;o__*Brocadiales*; f__*Scalinduaceae*;g__SCAELEC01
Rhodobiaceae bin1	3.1%	6.0	76	5,364	85.3	21	p__*Pseudomonadota*;c__*Alphaproteobacteria*;o__*Rhizobiales*; f__*Rhodobiaceae*;g__*Rhodobium*
Rhizobiaceae bin1	0.2%	5.9	674	5,184	86.5	20	p__*Pseudomonadota*;c__*Alphaproteobacteria*;o__*Rhizobiales*; f__*Rhizobiaceae*;g__RCIO01
Thalassobaculales bin1	0.2%	5.4	832	4,507	87.8	5.0	p__*Pseudomonadota*;c__*Alphaproteobacteria*; o__*Thalassobaculales*

Bin	Relative abundance (%)	Size	Contigs	CDSs	Completeness	Contamination	Phylogeny
Bradymonadaceae bin1	15%	3.0	48	2810	100	3.9	p__*Pseudomonadota*;c__*Gammaproteobacteria*; o__*Pseudomonadales*;f__*Porticoccaceae*;g__*Porticoccus*
Nitrosomonadaceae bin1	14%	3.1	89	2657	94.0	4.5	p__*Pseudomonadota*;c__*Gammaproteobacteria*; o__*Burkholderiales*;f__*Nitrosomonadaceae*;g__*Nitrosomonas*
Thalassospiraceae bin1	12%	4.6	45	4188	97.0	2.9	p__*Pseudomonadota*;c__*Alphaproteobacteria*; o__*Rhodospirillales*;f__*Thalassospiraceae*;g__*Thalassospira*
Rhodobacteraceae bin1	10%	4.0	34	3868	92.4	6.6	p__*Pseudomonadota*;c__*Alphaproteobacteria*; o__*Rhodobacterales*;f__*Rhodobacteraceae*;g__*Roseovarius*
Phycisphaerales bin1	5.5%	3.2	12	2788	86.6	3.1	p__*Planctomycetota*;c__*Phycisphaerae*;o__*Phycisphaerales*; f__*SM1A02*;g__GCA-2732755

Melioribacteraceae bin1	6.3%	4.7	96	3774	100	0	p__*Bacteroidota*;c__*Ignavibacteria*;o__*Ignavibacteriales*; f__*Melioribacteraceae*
Kapabacteriales bin1	5.8%	3.2	36	2770	100	0	p__*Bacteroidota*;c__*Kapabacteria*;o__*Kapabacteriales*; f__UBA2268;g__PGYR01
Balneolaceae bin1	4.8%	3.5	56	2891	100	0	p__*Bacteroidota*;c__*Rhodothermia*;o__*Balneolales*; f__*Balneolaceae*;g__*Balneola*
Alcanivoracaceae bin1	3.2%	4.7	54	4318	97.5	3.1	p__*Pseudomonadota*;c__*Gammaproteobacteria*; o__*Pseudomonadales*;f__*Alcanivoracaceae*;g__*Alcanivorax*
Oceanibaculaceae bin1	3.0%	3.7	32	3558	100	0	p__*Pseudomonadota*;c__*Alphaproteobacteria*; o__*Thalassobaculales*;f__*Oceanibaculaceae*;g__*Oceanibaculum*
Vicingaceae bin1	3.3%	3.4	41	2861	98.0	0.5	p__*Bacteroidota*;c__*Bacteroidia*;o__*Flavobacteriales*; f__*Vicingaceae*;g__BRH-c54
Marinicellaceae bin1	2.6%	3.0	22	2649	76.5	3.5	p__*Pseudomonadota*;c__*Gammaproteobacteria*; o__*Xanthomonadales*;f__*Marinicellaceae*;g__*Marinicella*
Bradymonadaceae bin1	1.3%	5.9	27	4870	100	0	p__*Myxococcota*;c__*Bradymonadia*;o__*Bradymonadales*; f__*Bradymonadaceae*;g__V1718
Rhodobacteraceae bin2	1.0%	4.4	33	4221	99.4	0.39	p__*Pseudomonadota*;c__*Alphaproteobacteria*;o__*Rhodobacterales*; f__*Rhodobacteraceae*
Rhizobiaceae bin2	1.0%	4.5	34	4341	88.7	11.6	p__*Pseudomonadota*;c__*Alphaproteobacteria*;o__*Rhizobiales*; f__*Rhizobiaceae*;g__STC3
Mycobacteriaceae bin1	0.9%	5.2	69	4855	98.4	0	p__*Actinobacteriota*;c__*Actinomycetia*;o__*Mycobacteriales*; f__*Mycobacteriaceae*;g__*Rhodococcus*
Flavobacteriaceae bin1	1.1%	3.1	38	2737	100	0.3	p__*Bacteroidota*;c__*Bacteroidia*;o__*Flavobacteriales*; f__*Flavobacteriaceae*;g__*Altibacter*

Dehalococcoidia bin1	0.9%	3.3	27	3247	90.8	6.4	p__*Chloroflexota*;c__*Dehalococcoidia*;o__UBA2979; f__UBA2979;g__W-Chloroflexi-9
Planctomycetaceae bin1	1.0%	8.2	61	6332	99.4	1.8	p__*Planctomycetota*;c__*Planctomycetes*;o__*Planctomycetales*; f__*Planctomycetaceae*;g__*Gimesia*
Phycisphaerales bin2	0.8%	3.9	89	3346	84.2	2.9	p__*Planctomycetota*;c__*Phycisphaerae*;o__*Phycisphaerales*; f__SM1A02;g__UBA6054
Parvibaculaceae bin1	1.0%	3.7	38	3561	99.9	2.2	p__*Pseudomonadota*;c__*Alphaproteobacteria*;o__*Parvibaculales*; f__*Parvibaculaceae*;g__Mf105b01
Sphingomonadaceae bin1	0.8%	3.9	59	3559	87.4	8.8	p__*Pseudomonadota*;c__*Alphaproteobacteria*; o__*Sphingomonadales*;f__*Sphingomonadaceae*;g__*Sphingopyxis*
Bradymonadia bin1	0.5%	9.7	385	6947	86.8	9.5	p__*Myxococcota*;c__*Bradymonadia*;o__UBA7976;f__UBA1532
Thermoleophilia bin1	0.5%	2.7	77	2652	96.6	1.3	p__*Actinobacteriota*;c__*Thermoleophilia*;o__Ga0077560; f__Ga0077560;g__Ga0077541
Rhizobiaceae bin3	0.6%	5.7	226	5353	96.4	13.3	p__*Pseudomonadota*;c__*Alphaproteobacteria*;o__*Rhizobiales*; f__*Rhizobiaceae*;g__*Nitratireductor*
Rhodospirillales bin1	0.5%	3.9	78	3528	77.7	7.9	p__*Pseudomonadota*;c__*Alphaproteobacteria*;o__*Rhodospirillales*; f__Casp-alpha2
Cyclobacteriaceae bin1	0.5%	5.7	636	4580	97.2	27.1	p__*Bacteroidota*;c__*Bacteroidia*;o__*Cytophagales*; f__*Cyclobacteriaceae*;g__ELB16-189
Rhizobiaceae bin4	0.4%	5.1	316	4655	87.1	12.3	p__*Pseudomonadota*;c__*Alphaproteobacteria*;o__*Rhizobiales*; f__*Rhizobiaceae*;g__RCIO01

Figure legends

Fig. S1. Dominance of the *Scalindua* sp. cells in the Percoll-separated *Scalindua* biomass. Planktonic *Scalinuda* sp. culture was maintained using a membrane bioreactor (panel a), and the buoyant density separation of the *Scalinuda* sp. cells was carried out to obtain a highly-enriched *Scalindua* biomass. In the anammox bacterial culture collected from the inside of the membrane bioreactor and the Percoll-separated *Scalindua* biomass, the *Scalindua* sp. cells accounted from *ca*. 90% and >98% of total cells (**panel b** and **c**, respectively) as determined by fluorescence *in-situ* hybridization analysis using the *Scalindua*-specific oligonucleotide Sca1129b probe. Scale bar corresponds to 100 μm.

Fig. S2 A calibration curve for calculating dissolved O_2 (DO) concentrations in the liquid media. Air was injected into the headspace of closed vials, and DO concentrations were determined after equilibrium at 25ºC for 12 h using an O_2 microsensor.

Fig. S3 Determination of dissolved O_2 (DO) concentration in the fluid media using an O_2 microsensor. A needle type O_2 microsensor (Unisense oxygen needle sensor OX-N 13621) was fixed using a clamp arm, and the vial was lifted up using a lab jack to reach the tip of the microsensor. One rotation (*i.e*., 360º rotation) of the handle knob on the jack move the vial upward with 3 mm of distance. Scale bar corresponds to 40 mm.

Fig. S4 The plastic pipette used for the collection of a core sample from the fluid media. The tip of the 10–mL plastic pipette (Φ7.5 mm) (Violamo, AsONE, Osaka, Japan) was cut, and the other top was connected to a disposable syringe. This pipette was inserted into the fluid media vertically, and collected core samples were dispensed into a 1.5 mL-plastic tubes. Scale bar corresponds to 50 mm.

Fig. S5 Anoxic urea degradation by the *Scalindua* biomass. The incubation was performed in duplicate, and the urea degradation found in each vial was shown in this figure. Ten mL of the Percoll-separated *Scalindua* biomass was anoxically (< 1 μM dissolved O_2) incubated in 20-mL glass vials at 25ºC in dark with the addition of 3 mM ^{14}N-urea and 3 mM $^{15}NO_2^-$ (**panel a** and **b**). The incubation was repeated with the addition of penicillin G (500 mg/L) (**panel c** and **d**) or without the *Scalindua* biomass (abiotic control) (**panel e** and **f**).

Fig. S6 Anoxic OCN^- degradation by the *Scalindua* biomass. The incubation was performed in duplicate, and the OCN^- degradation found in each vial was shown in this figure. Ten mL of the Percoll-separated *Scalindua* biomass was anoxically (< 1 μM dissolved O_2) incubated in 20-mL glass vials at 25ºC in dark with the addition of 3 mM $OC^{14}N^-$ and 3 mM $^{15}NO_2^-$ (**panel a** and **b**). The incubation was repeated with the addition of penicillin G (500 mg/L) (**panel c** and **d**) or without the *Scalindua* biomass (abiotic control) (**panel e** and **f**).

Fig. S7 Enrichment of aerobic ureolytic bacteria. Urea was fed into the Percoll-separated biomass, and the culture was incubated at 25ºC in dark under aerobic condition. After 32 d (indicated with a red arrow), urea was supplemented into the culture again. After 45 d, the culture was inoculated into a fresh media containing urea, and this subculturing was repeated 8 times in total. The black arrows indicated when the subculturing was carried out. The aerobic enrichment culture after 104 d of incubation was subjected to activity tests and genomic analyses.

Fig. S8 Vertical profiles of dissolved O_2 (DO) and NH_4^+ concentrations, and abundance of *Scalindua* 16S rRNA gene copy numbers in control experiments: a) abiotic incubation fed with ^{15}N-urea and $^{14}NO_2^-$, **b)** and **c)** incubation with ^{15}N-urea but without NO_2^-. In the panel **c)**, N_2 gas production in the headspace of the vials was examined, and no significant gas production occurred during the incubation.

Fig. S9 Bacterial community structure of the Percoll-separated *Scalindua* biomass and aerobic enrichment culture (left and right bars, respectively) as examined by 16S rRNA-gene amplicon sequencing analysis. Each bars represent specific bacterial phylum (left) or order/family (right), and the relative abundance of the 16S rRNA gene reads in total reads was shown.

Fig. S10 Metabolic potentials of metagenomic bins obtained from the Percoll-separated *Scalindua* biomass and the aerobic enrichment culture. The numbers on the top row corresponded to the metabolic modules shown on the bottom part of the figure, and the heatmap indicates metabolic pathway completeness.

Fig. S11 Amplicon sequencing analysis of *ureC* encoding catalytic subunit of urease. **a)** relative abundance of *ureC* reads obtained from the Percoll-separated *Scalindua* biomass and the aerobic enrichment culture. **b)** Primer-sequence mismatches between the used forward oligonucleotide L2F_V1 or reverse 733R primer and the *ureC* sequences found on the obtained *Rhizobiaceae*, *Nitrosomonadaceae*, and *Thalassospiraceae* bins. Degenerate bases are as following, Y for C or T, R for A or G, H for A, C or T, D for A, G, or T, and V for A, G, or C. The forward L2F_V1 primer had some mismatches, which likely resulted in over- or underestimation of the abundance of specific *ureC* reads.

a) Membrane bioreactor

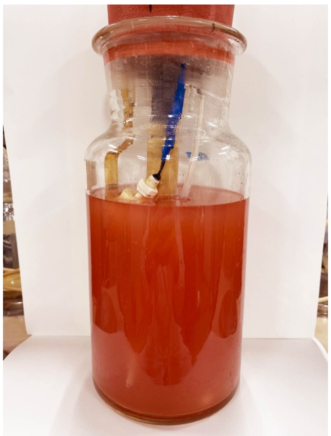

b) *Scalindua* culture collected from the membrane bioreactor

EUB-mix probe (FITC) Sca1129b probe (TRITC) EUB-mix & Sca1129 probes

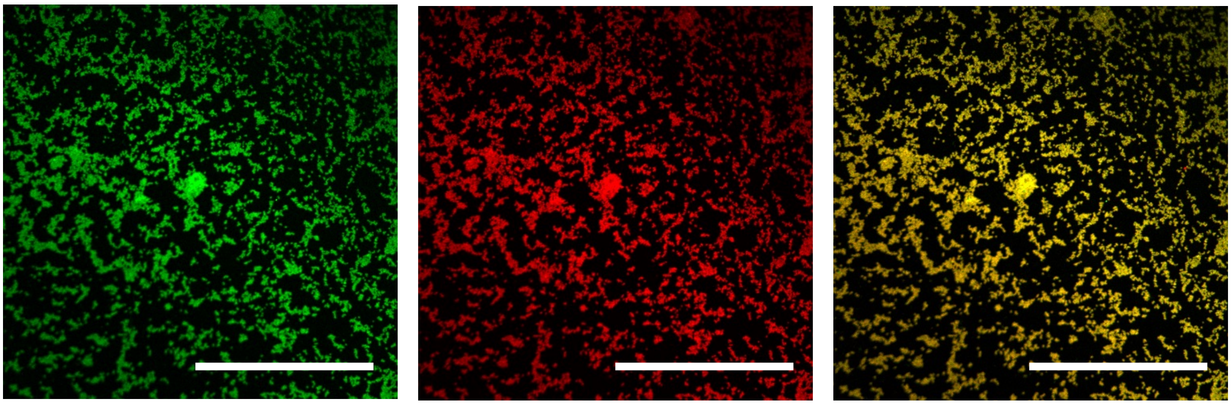

c) Percoll-separated *Scalindua* biomass

EUB-mix probe (FITC) Sca1129b probe (TRITC) EUB-mix & Sca1129 probes

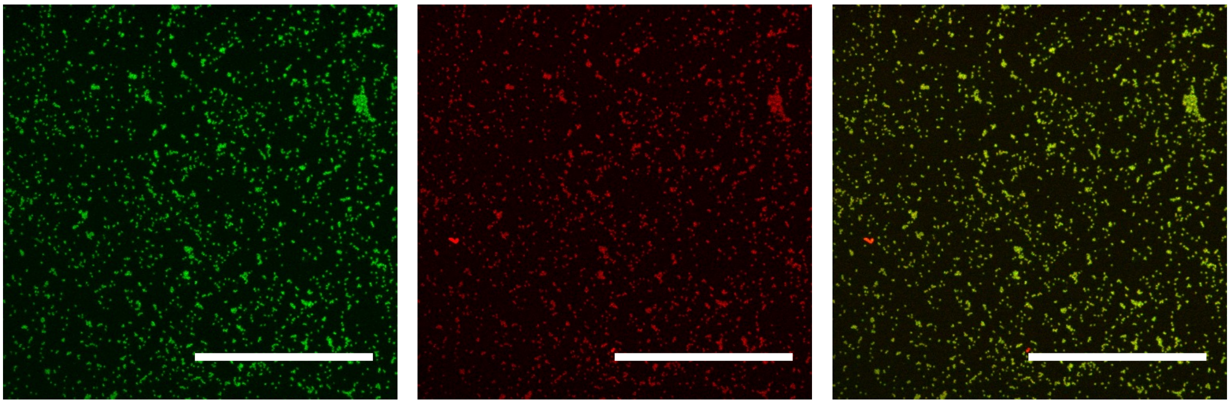

Fig. S1 (Oshiki *et al.*)

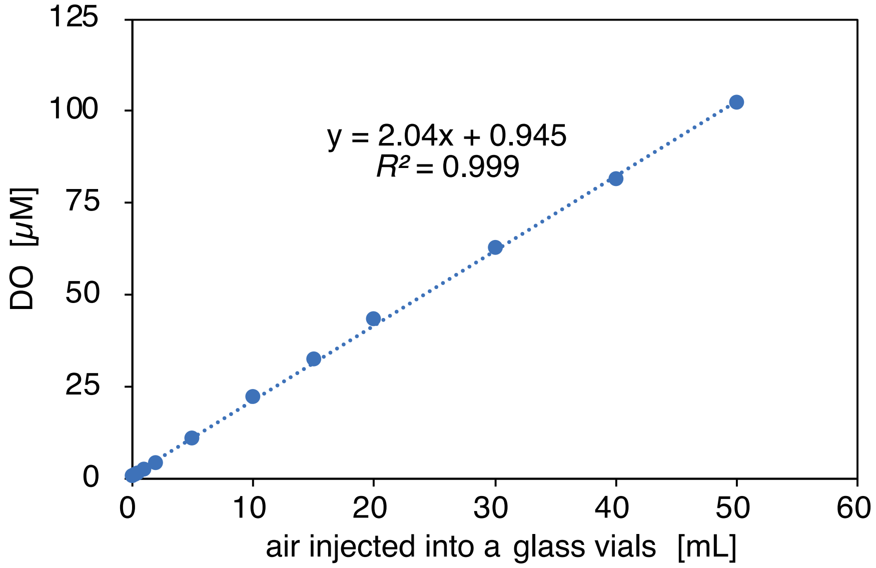

Fig. S2 (Oshiki *et al.*)

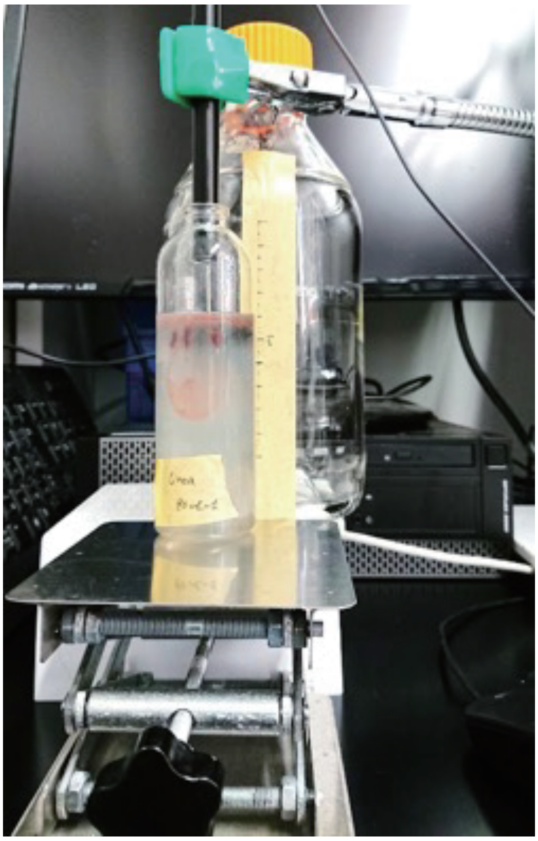

Fig. S3 (Oshiki *et al.*)

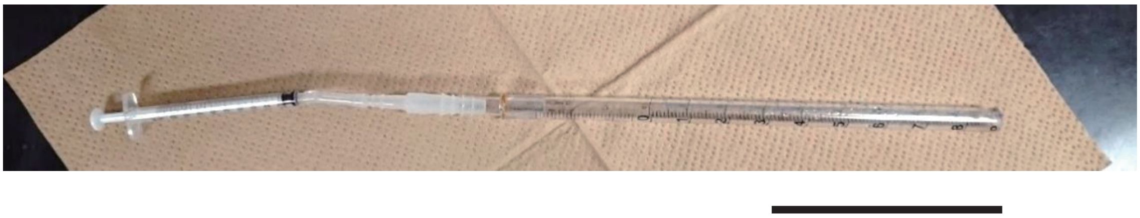

Fig. S4 (Oshiki *et al.*)

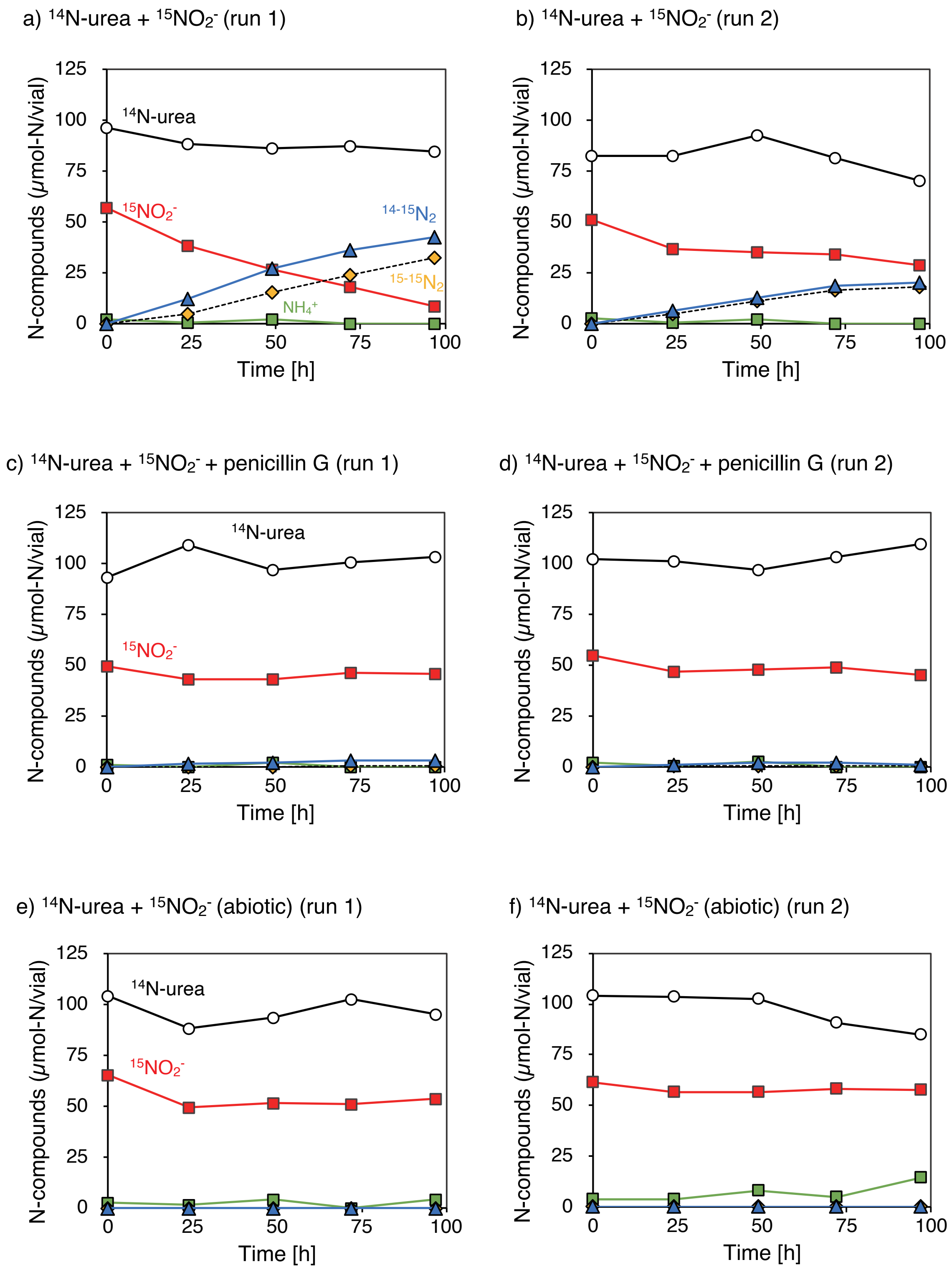

Fig. S5 (Oshiki *et al.*)

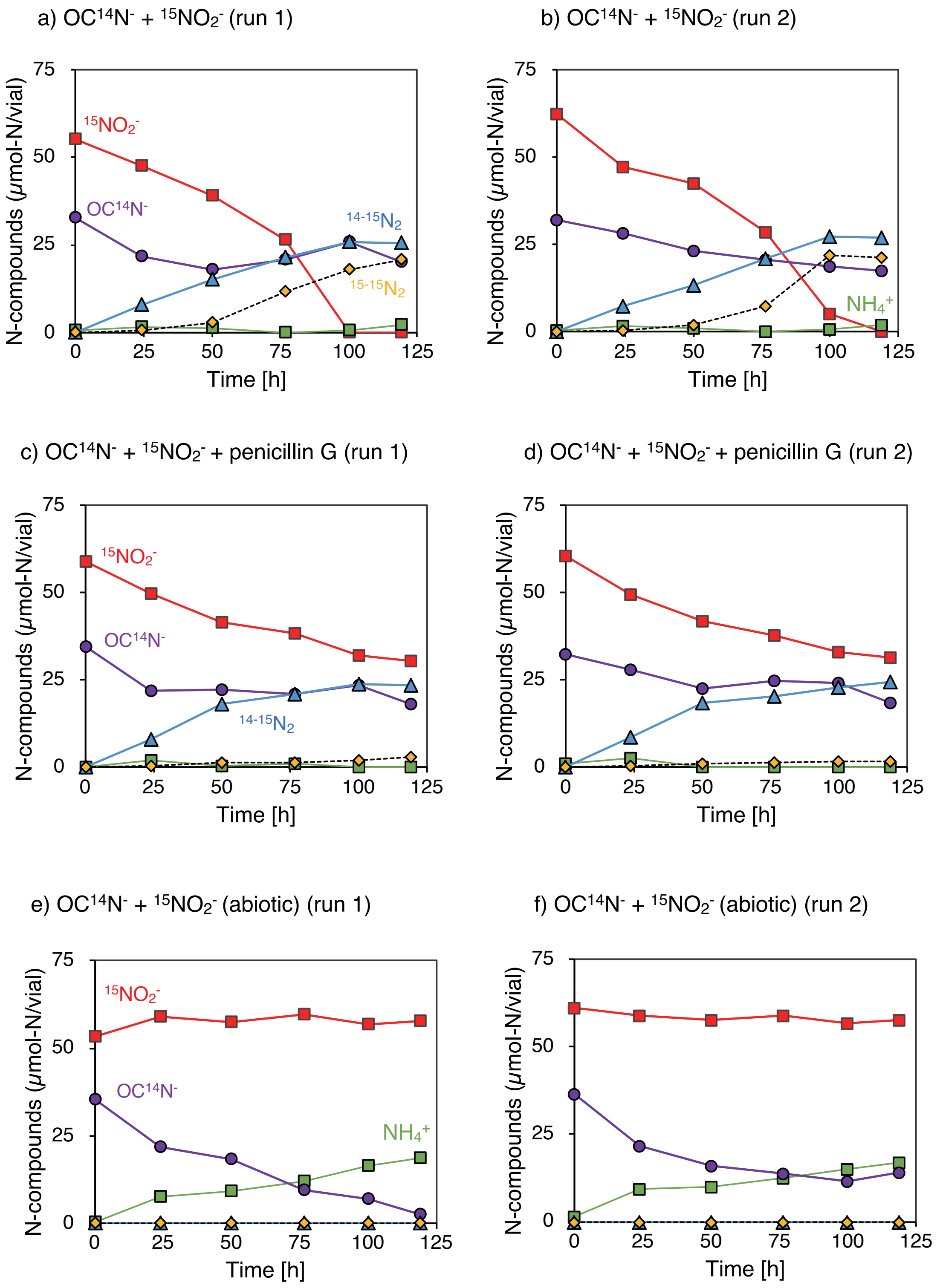

Fig. S6 (Oshiki *et al.*)

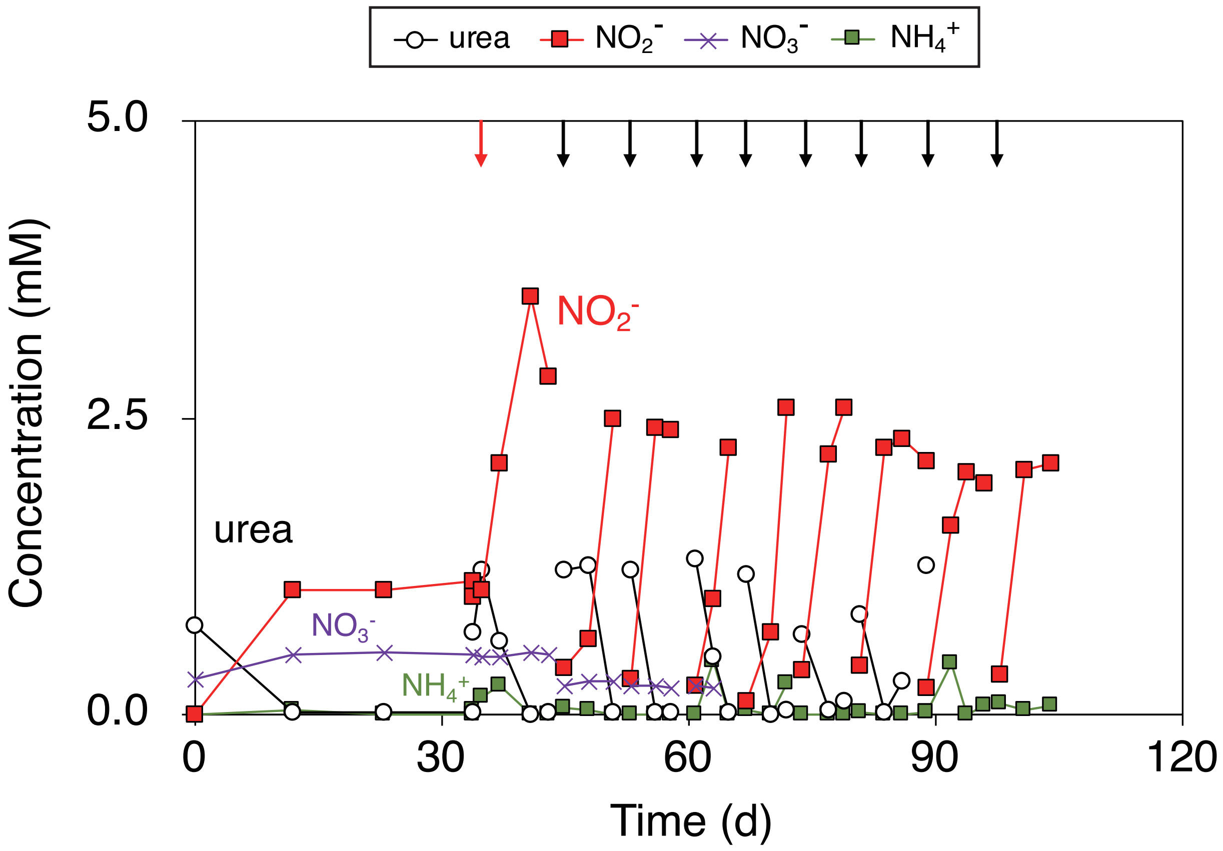

Fig. S7 (Oshiki *et al.*)

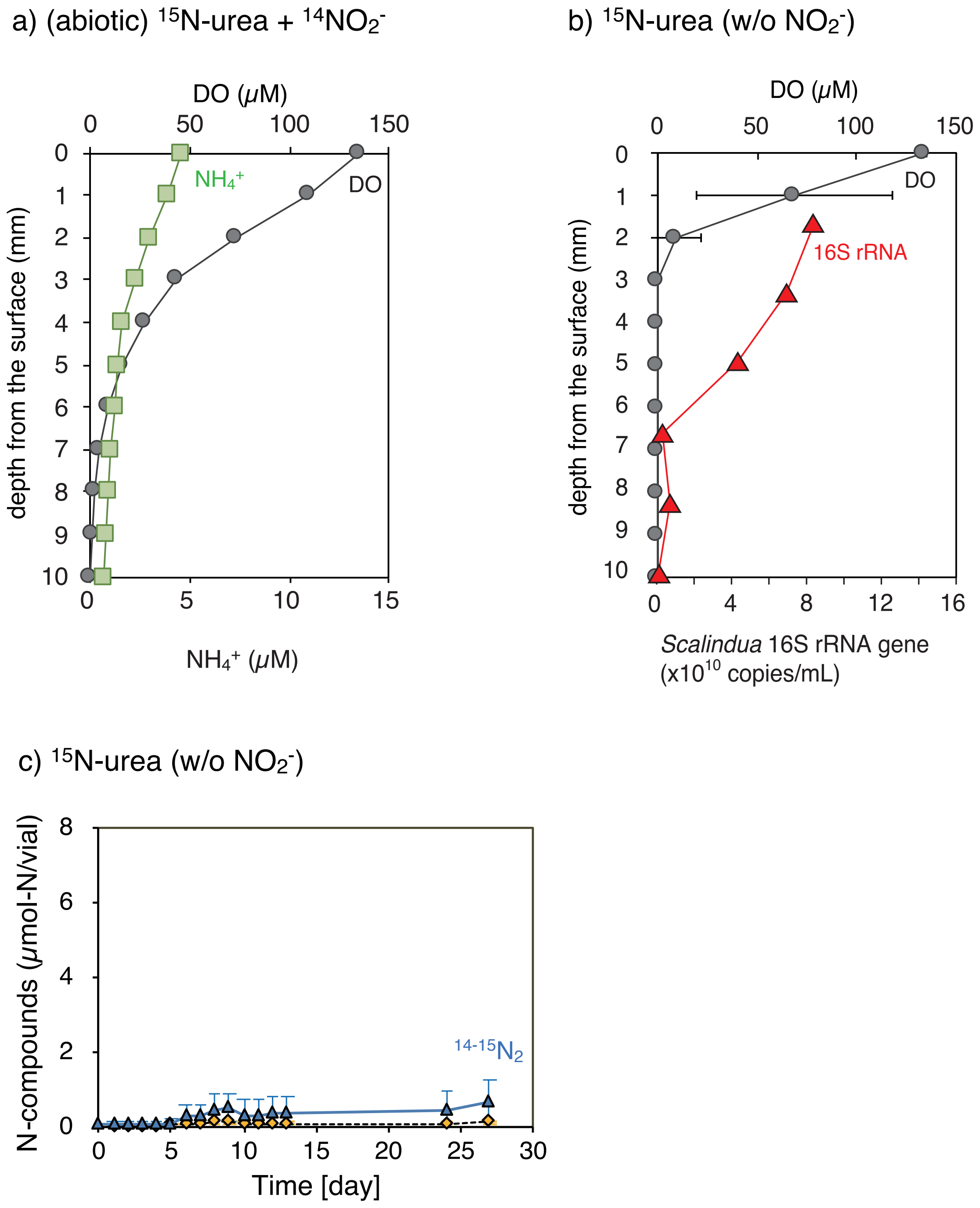

Fig. S8 (Oshiki *et al.*)

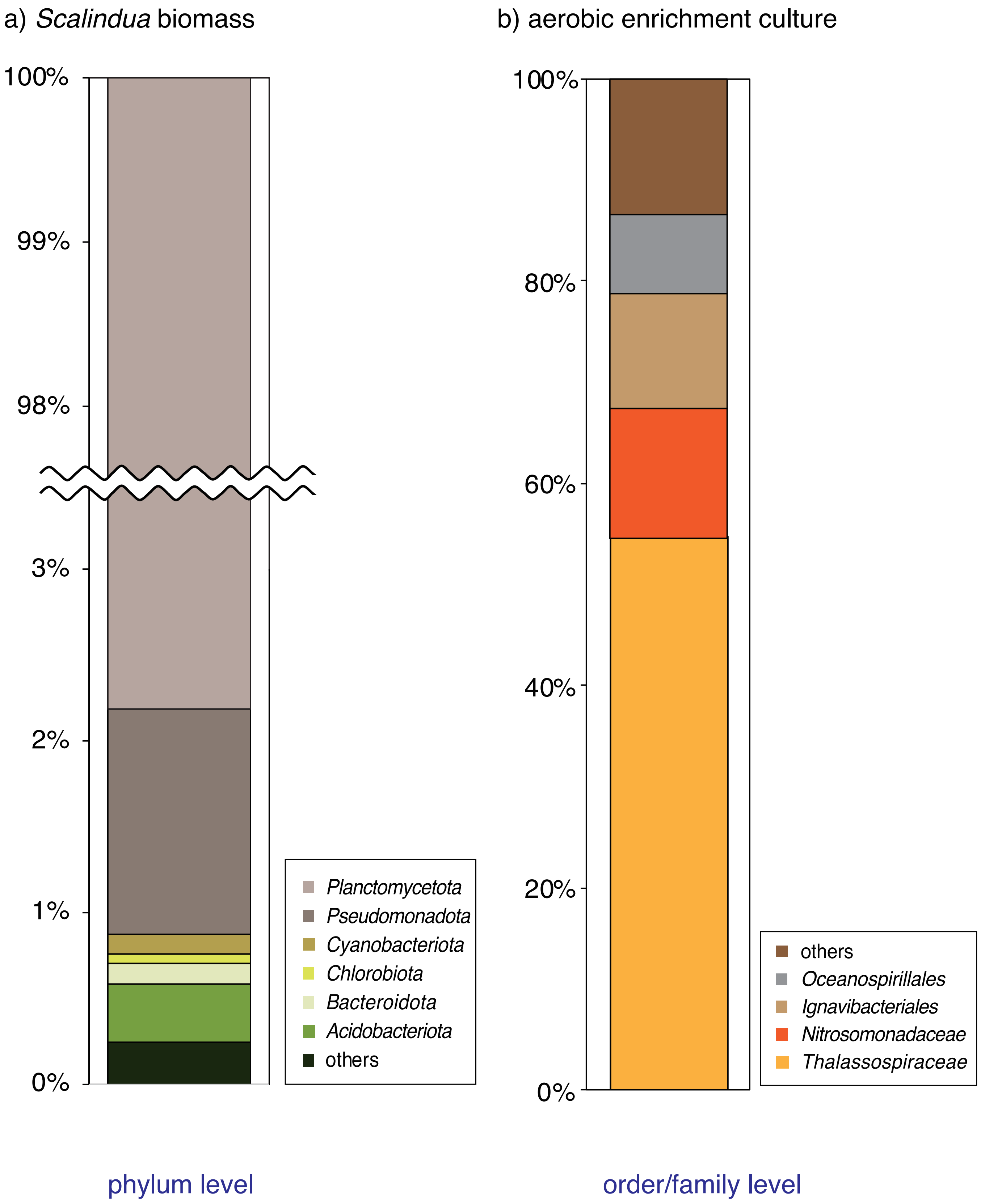

Fig. S9 (Oshiki *et al.*)

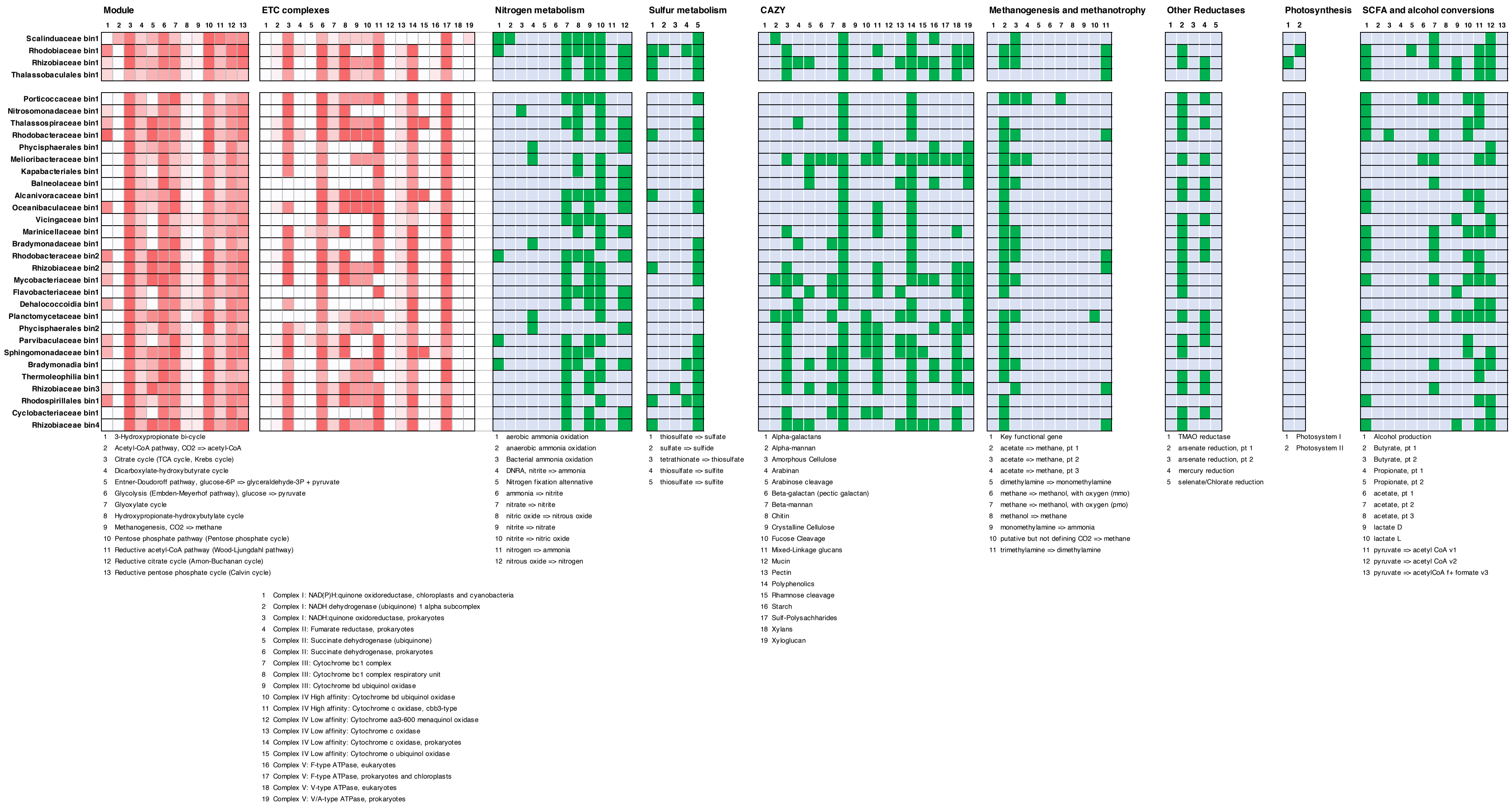

Fig. S10 (Oshiki *et al.*)

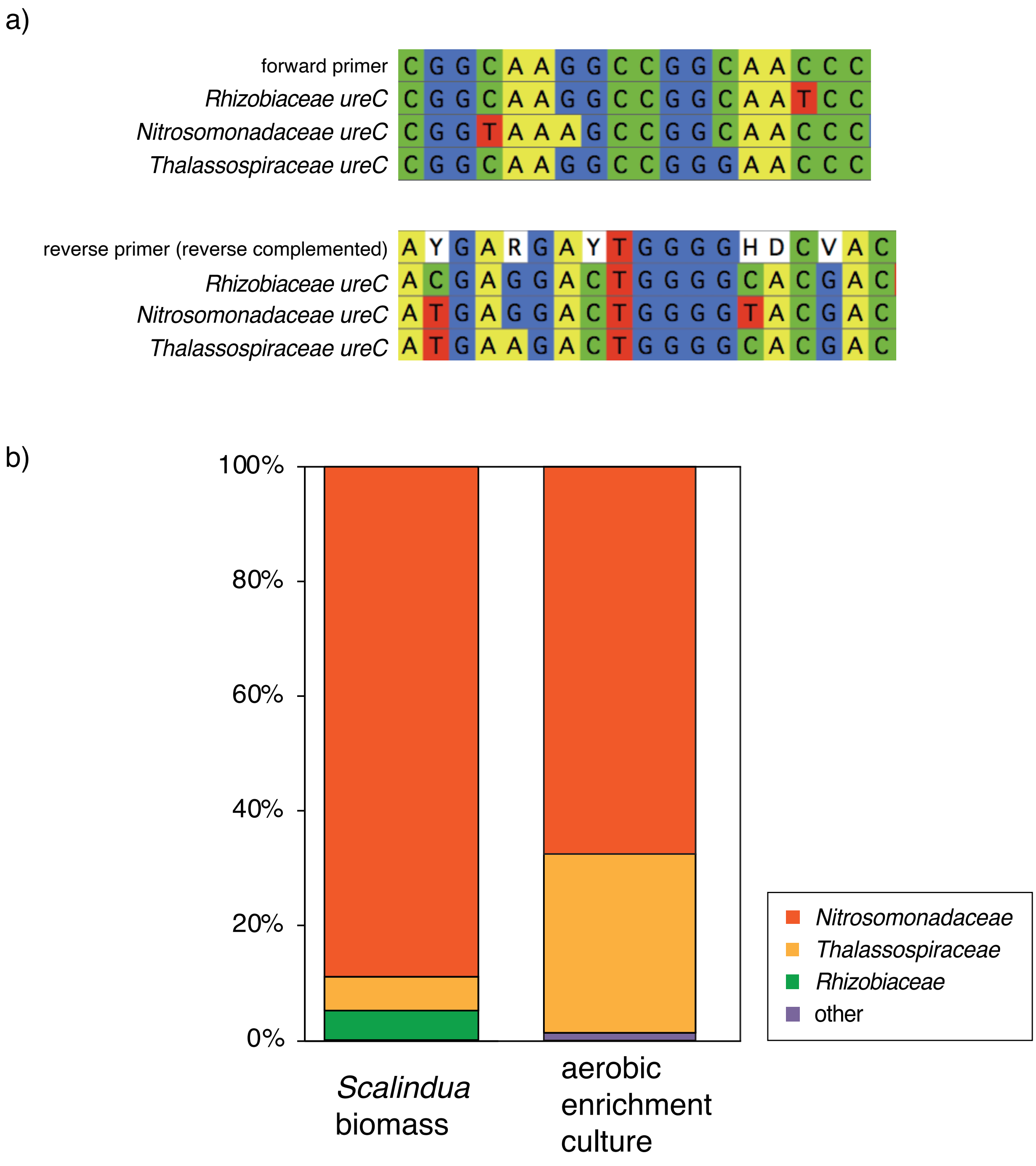

Fig. S11 (Oshiki *et al.*)
